# An Active Fraction of *Trillium tschonoskii* Promotes the Regeneration of Intestinal Epithelial Cells After Irradiation

**DOI:** 10.3389/fcell.2021.745412

**Published:** 2021-11-02

**Authors:** Feiling Song, Sihan Wang, Xu Pang, Zeng Fan, Jie Zhang, Xiaojuan Chen, Lijuan He, Baiping Ma, Xuetao Pei, Yanhua Li

**Affiliations:** ^1^Experimental Hematology and Biochemistry Lab, Beijing Institute of Radiation Medicine, Beijing, China; ^2^Stem Cells and Regenerative Medicine Lab, Institute of Health Service and Transfusion Medicine, Beijing, China; ^3^South China Research Center for Stem Cell & Regenerative Medicine, SCIB, Guangzhou, China; ^4^Department of Pharmaceutical Sciences, Beijing Institute of Radiation Medicine, Beijing, China

**Keywords:** intestinal epithelium, active fractions of *Trillium tschonoskii*, intestinal organoid, radiation, regeneration

## Abstract

Despite significant scientific advances toward the development of safe and effective radiation countermeasures, no drug has been approved for use in the clinic for prevention or treatment of radiation-induced acute gastrointestinal syndrome (AGS). Thus, there is an urgent need to develop potential drugs to accelerate the repair of injured intestinal tissue. In this study, we investigated that whether some fractions of Traditional Chinese Medicine (TCM) have the ability to regulate intestinal crypt cell proliferation and promotes crypt regeneration after radiation. By screening the different supplements from a TCM library, we found that an active fraction of the rhizomes of *Trillium tschonoskii* Maxim (TT), TT-2, strongly increased the colony-forming ability of irradiated rat intestinal epithelial cell line 6 (IEC-6) cells. TT-2 significantly promoted the proliferation and inhibited the apoptosis of irradiated IEC-6 cells. Furthermore, in a small intestinal organoid radiation model, TT-2 promoted irradiated intestinal organoid growth and increased Lgr5^+^ intestinal stem cell (ICS) numbers. More importantly, the oral administration of TT-2 remarkably enhanced intestinal crypt cell proliferation and promoted the repair of the intestinal epithelium of mice after abdominal irradiation (ABI). Mechanistically, TT-2 remarkably activated the expression of ICS-associated and proliferation-promoting genes and inhibited apoptosis-related gene expression. Our data indicate that active fraction of TT can be developed into a potential oral drug for improving the regeneration and repair of intestinal epithelia that have intestinal radiation damage.

## Introduction

A high dosage of ionizing radiation in pelvic and abdominal cancer radiotherapy or radiological emergency scenarios can cause acute gastrointestinal syndrome (AGS) in those affected (Booth et al., 2012; Chapel et al., 2013). Under irradiation, epithelial cells in intestinal villi and crypts are easily injured; this causes cell apoptosis or death. As the gastrointestinal epithelial integrity is destroyed, patients with AGS suffer from different degrees of nausea, vomiting, disturbance of water and electrolytes, loss of the intestinal immune barrier, bacteremia and sepsis, and even death (Shadad et al., 2013). Thus, it is essential to use radiation countermeasure agents to ameliorate AGS symptoms or rescue patients. However, so far, there is no effective drug approved for application in the clinical setting for prevention and treatment of AGS.

Several labs have been devoted to develop effective radiation protectants or mitigators. A variety of agents that function differently, including blockers of oxygen consumption, free radical scavengers, somatostatin analogs, growth factors, TLR5 agonists, endothelial protection drugs, vitamin E analogs, and Chinese herbal medicine have shown protective or mitigative effects in animal models with multiple types of intestinal radiation damage (Berbee and Hauer-Jensen, 2012; Shadad et al., 2013; Choi et al., 2019). However, it remains an unmet target to develop radiation countermeasure drugs to accelerate the regeneration of radiation-injured intestinal tissue. *In vitro* drug screening systems based on cell lines or primary cells are often used for drug discovery (Ayehunie et al., 2018; Dutton et al., 2019). The rat intestinal crypt cell line, rat intestinal epithelial cell line 6 (IEC-6), is a good cell model for the discovery of new chemical compound with the ability to mitigate the effects of radiation-induced intestinal epithelial damage (Wang S. et al., 2020). The stem cell population in intestinal crypts is responsible for intestinal villi renewal and regeneration after injury *in vivo*. This process is mainly driven by intestinal crypt base columnar cells, which are active, fast-cycling intestinal stem cells (ISCs) marked by Lgr5, CD133, and Sox9 expression (Rizk and Barker, 2012; Roche et al., 2015). Recently, using a three-dimensional extracellular matrix culture system, ISCs were cultured to grow into self-organizing mini-gut structures, e.g., intestinal organoids (Sato and Clevers, 2013; Sugimoto and Sato, 2017; Wallach and Bayrer, 2017; Rahmani et al., 2019). The intestinal organoid culture technique provides a real intestinal epithelium model to evaluate the effect of radiation countermeasure agents (Kim et al., 2020; Martin et al., 2020).

Some Traditional Chinese Medicine (TCM) have been reported to play a protective or mitigative role in radiation-induced intestinal toxicity (Kim et al., 2002; Liu et al., 2006; Dutta et al., 2012; Rockwell et al., 2013; Yang et al., 2019). It is valuable and meaningful to find more effective TCM that can modulate intestinal epithelial cell function. Hence, we performed a preliminary screening experiment using an intestinal crypt cell line (IEC-6) culture and colony-forming assays after irradiation to evaluate the function of some TCM and their fractions collected in our lab. Notably, we found that an active fraction of the rhizomes of *Trillium tschonoskii* Maxim (TT), strongly increased the colony-forming ability of irradiated IEC-6 cells. TT is a perennial herb plant distributed in most areas of central and western mainland China (Wang B. et al., 2020). The rhizomes of this plant are used as a folk medicine (“Yan-lingcao”) for the treatment of neurasthenia, cancer, headache, and some inflammatory diseases in the clinic (Wang S. et al., 2020). Here, we firstly reported that an active fraction of TT with the ability to decrease the apoptosis and enhance the proliferation of irradiated intestinal epithelia *in vitro*. Using an *in vitro* intestinal organoid culture system and a high-dose abdominal irradiation (ABI) model, we characterized the role of the active fraction of TT in intestinal epithelia repair following radiation injury. Herein, we provide scientific evidence for the use of the active fraction of TT as a potential drug to ameliorate AGS.

## Materials and Methods

### Separation and Extraction of the Fractions of *Trillium tschonoskii*

Rhizomes of TT (collected from the Shennongjia, Hubei province; 3 kg) were crushed and extracted using 50% EtOH with three refluxes (24 L, 24 L, 24 L, each for 2 h). The filtered solution was concentrated *in vacuo* and centrifugated to obtain the supernatant and sediment. The supernatant was passed through an SP825 macroporous resin column (column volume 6 L) and eluted using a gradient condition of EtOH-H_2_O (*v*/*v*, 0:100 → 15:85 → 30:70 → 50:50 → 75:25 → 95:5, three column volumes per concentration) to yield six fractions (TT-1 to TT-6). TT-2 (15% EtOH elution) was lyophilized to yield 20.0 g powder, and half of TT-2 was dissolved in water and then subjected to an HP20 macroporous resin column (column volume 2 L) and eluted using a gradient condition of EtOH-H_2_O (v/v, 0:100 → 15:85 → 50:50, three column volumes per concentration) to yield three fractions named TT-2-0 (8.1 g), TT-2-15 (1.1 g), and TT-2-50 (0.5 g).

### Cells, Mice, and Radiation Treatment

IEC-6 cells were obtained from the American Type Culture Collection (ATCC) and cultured in Dulbecco’s Modified Eagle Medium (DMEM) supplemented with 10% fetal bovine serum (FBS) at 37°C and 5% (v/v) CO_2_ under a humidified atmosphere. The cells were irradiated at a rate of 54.22 cGy/min using a ^60^Co irradiator with a total dose of 10 Gy. The irradiated cells were cultured in a medium with or without the addition of TT-2 (10 μg/mL), and TT-2 was dissolved in water to configure different concentrations.

C57BL/6 mice were obtained from a commercial vendor (Beijing Vital River Laboratory Animal Technology Co., Ltd.) and housed under standardized conditions with controlled temperature and humidity and a 12/12-h day/night light cycle for 7 days. The mice were anesthetized by intraperitoneal injection of pentobarbital, and then the abdomen part or mice were exposed to irradiation (non-abdomen body parts including the skeleton were shielded with lead blocks) at a rate of 54.22 cGy/min using a ^60^Co irradiator (Beijing Institute of Radiation Medicine, Beijing, China) with a total dose of 14 Gy. For oral delivery, TT-2 was dissolved in water at a concentration of 10 mg/kg and administered by gavage every day from day −2 to 4. C57BL/6 mice were exposed to 14 Gy ABI on day 0. After irradiation, 200 μL water with or without TT-2 was delivered by gavage to each mouse. The irradiated mice were kept in sterile water containing antibiotics after irradiation.

Irradiated IEC-6 cells (∼1,000) were seeded in six-well plates and incubated for 7 days with or without fractions derived from a TCM bank collected in our lab. After the colonies were fixed and stained with crystal violet, colony numbers were counted. Three repeated experiments were carried out in each group.

### 5-Ethynyl-2′-Deoxyuridine Incorporation and Detection

The EdU assay was performed using an EdU Kit (RiboBio, C10310-1). Irradiated IEC-6 cells were cultured with or without TT-2 (10 μg/mL) for 48 h in DMEM supplemented with 10% FBS, and then switched to fresh DMEM supplemented with EdU (50 μM) and incubated for 2 h; this was followed by fixation, permeabilization, and EdU staining with Apollo567 (RiboBio, C00031). The nuclei were stained with DAPI, and the staining of EdU-positive cells was observed using fluorescent reverse microscopy (UltraVIEW VOX, PerkinElmer). The percentage of incorporation of EdU in the nucleus of irradiated IEC-6 cells in each field was calculated and expressed as the mean ± SD. For flow cytometry assays, the cells were exposed to 10 μM EdU for 2 h at 37°C, and were prepared and treated using a Click-iT^TM^ Plus EdU Alexa Fluor^TM^ 647 Flow Cytometry Assay Kit (C10635, Thermo Fisher Scientific) according to the manufacturer’s instructions. Flow cytometry analysis was performed using the FACSCalibur platform (BD Biosciences) to detect EdU incorporation.

### Apoptosis Assays

Irradiated IEC-6 cells were cultured with or without TT-2 (10 μg/mL) in DMEM supplemented with 10% FBS. After 48 h of incubation, an apoptosis detection kit (AD10, DOJINDO) was used according to the manufacturer’s instructions to test the apoptosis rate. In brief, after the cells were collected and washed with PBS, then were labeled with Annexin V [Fluorescein Isothiocyanate (FITC)] and PI Solution, and incubated for 15 min in the dark at room temperature. Then the apoptosis rate was measured in a flow cytometer. All the experiments were repeated three times.

TUNEL staining was carried out according to the manufacturer’s instructions (Promega). The mouse intestinal slides were incubated with proteinase K for 20 min at room temperature and washed with PBS, then incubated in TdT buffer containing TUNEL reaction mixture at 37°C for 1 h. Afterward, the slides were counterstained with DAPI to label DNA and thus the cell nucleus and mounted for fluorescent microscopy with Nikon fluorescence microscope. Quantitation analysis of the TUNEL assay results was done by counting DAPI-staining cell numbers and the TUNEL-positive cell numbers in the intestinal crypts and villi to calculate the apoptotic cells. There are 3 mice in each group, and at least 30 villi or crypts in the small intestine were counted for the number of positive cells.

### qRT-PCR Assays

Cells were collected and total RNA was isolated using TRIzol reagent (15596018, Invitrogen) according to the manufacturer’s instructions. Then, 800 ng of total RNA was reverse-transcribed into cDNA. Real-time quantitative PCR analysis was performed on a Bio-Rad CFX Connect Real-time System thermocycler using the SYBR Green PCR Master Mix (TaKaRa). The RNA levels were normalized using HPRT or GAPDH as an internal control. All the experiments were repeated three times. The primers are shown in detail in Supplementary Table 1.

### Crypt Isolation and Organoid Culture

The small intestines of mice were opened longitudinally and washed repeatedly with cold PBS (containing 100 U/mL or 100 μg/mL penicillin/streptomycin) until there were no visible impurities. The tissue was cut into 2–3 mm pieces, which were placed in cold 2.5 mM EDTA (AM9261, Invitrogen) at 4°C for 30 min. After removal of the EDTA medium, the tissue fragments were shaken to release crypts and then passed through a 70 μm cell strainer to remove the remaining villi. Isolated crypts were washed with cold PBS containing 0.1% BSA (A3311, Sigma) and centrifuged (∼290 × *g*) 10 times for enrichment. The crypts were buried in 30 μL cold Matrigel^®^ matrix (356231, Corning) and 30 μL IntestiCult^TM^ Organoid Growth Medium (06005, STEMCELL) at a density of 200 crypts per well. Fresh medium was replaced every 3 days.

### Hematoxylin and Eosin Staining Assays

Paraffin sections of the small intestine were melted at 56°C for 30–60 min and hydrated in 100, 90, and 70% ethanol for 5 min. The sections were immersed in Mayer hematoxylin (ZLI-9610, Zsbio) for 5 min without differentiation, and then immersed in acidified eosin ethanol solution (ZLI-9613, Zsbio) for 2–5 min. The length of villus or crypt in each group was determined by counting 30 intact villi or crypts and reported as the mean ± SD. Three mice were used in each group.

### Immunohistochemistry Assays

Paraffin sections were incubated overnight with primary antibodies in 1% horse serum albumin (PK-6200, Vector) at 4°C. The next day, the samples were washed with PBS and incubated with secondary antibodies at 25°C for 1 h. After washing with PBS, the sections were developed using ImmPACT^®^ NovaRED^®^ Substrate, Peroxidase (HRP) (SK-4805, Vector) for color reaction, and then observed under a microscope. The number of positive cells in 30 complete crypts was counted and expressed as the mean ± SD. Three mice were used in each group. The antibodies used were anti-BrdU (5292, CST), anti-Ki67 (9129, CST), anti-Cyclin D1 (2978, CST), anti-Lgr5/GPR49 (MAB8240, R&D Systems), anti-Sox9 (82630, CST), anti-Lysozyme (ab108508, Abcam), anti-Chromogranin A (GTX113165, GeneTex), and anti-Muc2 (GTX100664, GeneTex).

### *In situ* Hybridization

A small section of the intestine was embedded and made into paraffin sections. Then the RNA scope *in situ* hybridization 2.5 HD red detection kit (Advanced Cell Diagnostic, Newark, CA, United States) was used according to the manufacturer’s instructions to process the intestinal sections. In brief, the intestinal tissue underwent target retrieval, permeabilization, hybridization of Lgr5 (MAB8240, R&D Systems), amplification, and visualization using DAB-A and DAB-B. And then the intestinal sections were observed under a microscope. The expression of Lgr5 was quantitatively analyzed according to the five-grade scoring system recommended by the manufacturer (0, no staining; 1, 1–3 dots/cell; 2, 4–10 dots/cell; 3, >10 dots/cell; 4, >15 dots/cell with >10% of dots in clusters). The H-score was calculated as: (% of grade 1 cells × 1) + (% of grade 2 cells × 2) + (% of grade 3 cells × 3) + (% of grade 4 cells × 4). In addition, a cell with one or more dots was regarded as Lgr5-positive. Three mice were used in each group.

### Fluorescein Isothiocyanate-Dextran Test

C57BL/6 mice were orally administrated with 200 μL water with or without 10 mg/kg TT-2 from day −2 to 4. These mice were exposed to 14 Gy ABI on day 0. Four days after irradiation, FITC-Dextran (Sigma-Aldrich, St. Louis, MO, United States) was administered to the mice by oral gavage at a concentration of 0.6 mg/g body weight and a volume of 20 μL. Four hours after gavage, the serum of mice was collected, and 50 μL of both diluted serum samples and standards as well as blanks (PBS and diluted serum from untreated animals), were transferred to black 96-well microplates. FITC-Dextran concentrations were analyzed with a fluorescence spectrophotometer and fluorescence intensity was measured (excitation, 492 nm; emission, 525 nm).

### Statistical Analysis

Data were expressed as means ± SD. The paired *t*-test was used to determine the statistical significance between the two groups. One-way ANOVA followed by Dunnett’s *post hoc* test was used to compare the means of three or more independent groups. Results with *p* < 0.05 were considered statistically significant.

## Results

### Active Fraction of *Trillium tschonoskii* Increases Colony-Forming Ability of Irradiated IEC-6 Cells

**FIGURE 1:**
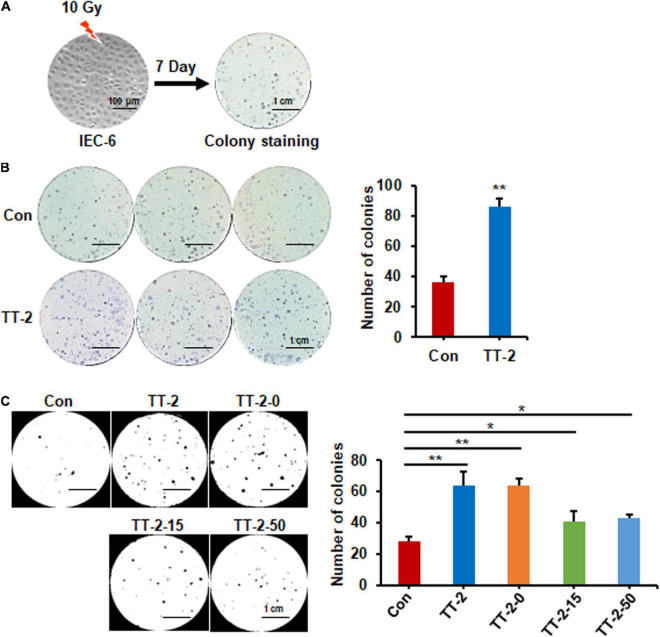
Active fraction of *Trillium tschonoskii* (TT) promoted colony formation of rat intestinal epithelial cell line 6 (IEC-6) cells. **(A)** Schematic diagram of colony formation by IEC-6 cells after 10 Gy irradiation. **(B)** Representative colony image and colony numbers formed by IEC-6 cells after 10 Gy irradiation and addition of H_2_O, or TT-2 to the culture medium (**p < 0.01, scale bar = 1 cm). **(C)** Representative colony staining image and colony numbers formed by IEC-6 cells after 10 Gy irradiation and addition of TT-2 or samples isolated after elution with different concentrations of ethanol (TT-2-0, TT-2-15, and TT-2-50) (*p < 0.05, **p < 0.01, scale bar = 1 cm).

To discover traditional Chinese herbs that may enhance intestinal epithelial repair after irradiation, we firstly chose a TCM library to screen potential effective fractions by performing the colony-forming assay of IEC-6 cells after 10 Gy irradiation (Figure 1A). We found that over 10 fractions from the TCM library increased the number of colonies formed by irradiated IEC-6 cells (data not shown). Notably, the active fractions of *Trillium tschonoskii* extracts (TT) numbered TT-2 caused 1.4-fold increases in the number of colonies formed by irradiated IEC-6 cells (Figure 1B). TT-2 was then isolated into three kinds of active fractions after elution with different concentrations of ethanol (0, 15, and 50%), these were named TT-2-0, TT-2-15, and TT-2-50. All these active fractions significantly increased the number of colonies formed by irradiated IEC-6 cells (Figure 1C). TT-2 and TT-2-0 were the same fraction from the TCM, and showed a better effect on increasing the colony-forming ability of irradiated IEC-6 cells than TT-2-15 and TT-2-50. These results indicated that the active fractions of TT might have the ability to promote intestinal cell regeneration after irradiation.

### Active Fraction of *Trillium tschonoskii* Promotes Proliferation and Inhibits Apoptosis of Irradiated IEC-6 Cells

**FIGURE 2:**
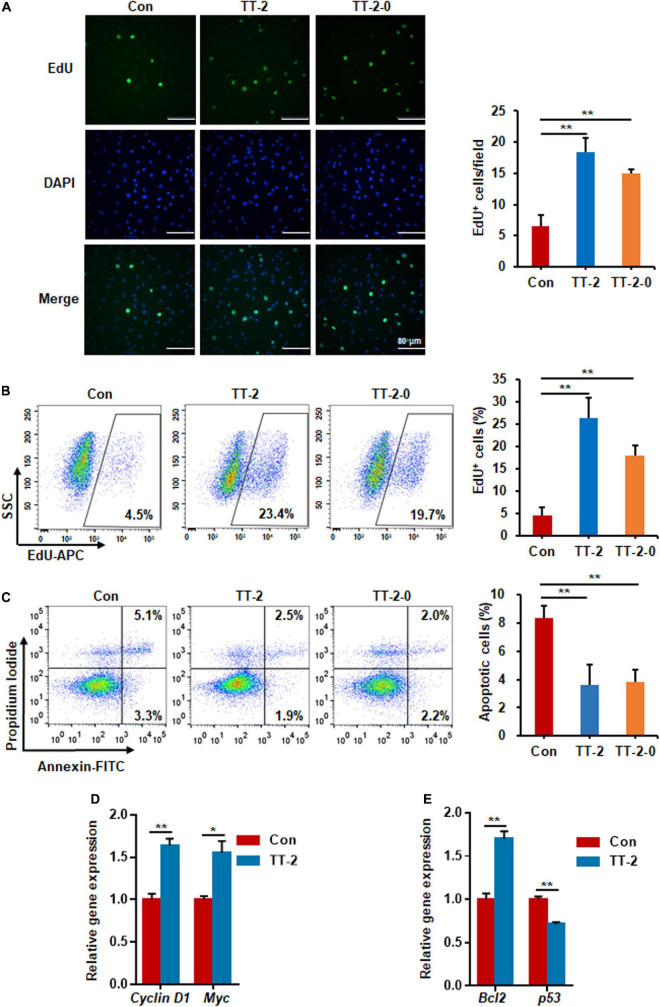
Active fraction of *Trillium tschonoskii* (TT) promoted proliferation and inhibited apoptosis of IEC-6 cells after irradiation. **(A)** Immunostaining analysis of EdU in IEC-6 cells after irradiation with or without TT-2 or TT-2-0 for 48 h (**p < 0.01, scale bar = 80 μm). **(B)** Percentage analysis of EdU incorporation for 4 h in IEC-6 cells after 10 Gy irradiation with or without TT-2 or TT-2-0 (**p < 0.01). **(C)** Percentage of apoptotic cells among IEC-6 cells after 10 Gy irradiation with or without TT-2 or TT-2-0 treatment for 48 h (**p < 0.01). **(D)** qPCR for proliferation-related gene expression in IEC-6 cells after 10 Gy irradiation with or without TT-2 treatment for 48 h (*p < 0.05, **p < 0.01). **(E)** qPCR for apoptosis-related gene expression in IEC-6 cells after 10 Gy irradiation with or without TT-2 treatment for 48 h (*p < 0.05, **p < 0.01).

We performed EdU incorporation experiments to further evaluate whether the active fraction of TT could directly regulate the proliferation of intestinal crypt cells after irradiation. The results of immunostaining of incorporated EdU showed that TT-2 and TT-2-0 notably increased the incorporation of EdU in the nucleus of irradiated IEC-6 cells compared to that in the control group (Figure 2A). Flow cytometry data of EdU-incorporated cell percentages also demonstrated a similar role of TT-2 and TT-2-0 in enhancing incorporation (Figure 2B). Next, we assessed the apoptosis rate of irradiated IEC-6 cells after TT-2 or TT-2-0 treatment for 48 h. The apoptotic cell percentage of irradiated cells was significantly lower in the TT-2 or TT-2-0 treatment group than in the controls (Figure 2C). Given that TT-2 and TT-2-0 were the same active fraction from TT, we chose TT-2 to further evaluate its role in proliferation- and apoptosis-related gene expression. The qPCR results demonstrated that TT-2 treatment significantly enhanced the expression of the proliferation-related genes, *Cyclin D1* and *Myc* (Figure 2D). The expression levels of the anti-apoptosis-related gene, *Bcl-2*, significantly increased after TT-2 treatment. In contrast, the expression of the *p53* gene was inhibited by TT-2 (Figure 2E). These results indicated that TT-2 might have enhanced intestinal crypt cell regeneration mainly by regulating irradiated crypt cell proliferation and inhibiting cell apoptosis.

### *Trillium tschonoskii*-2 Promotes Intestinal Organoid Growth After Irradiation

**FIGURE 3:**
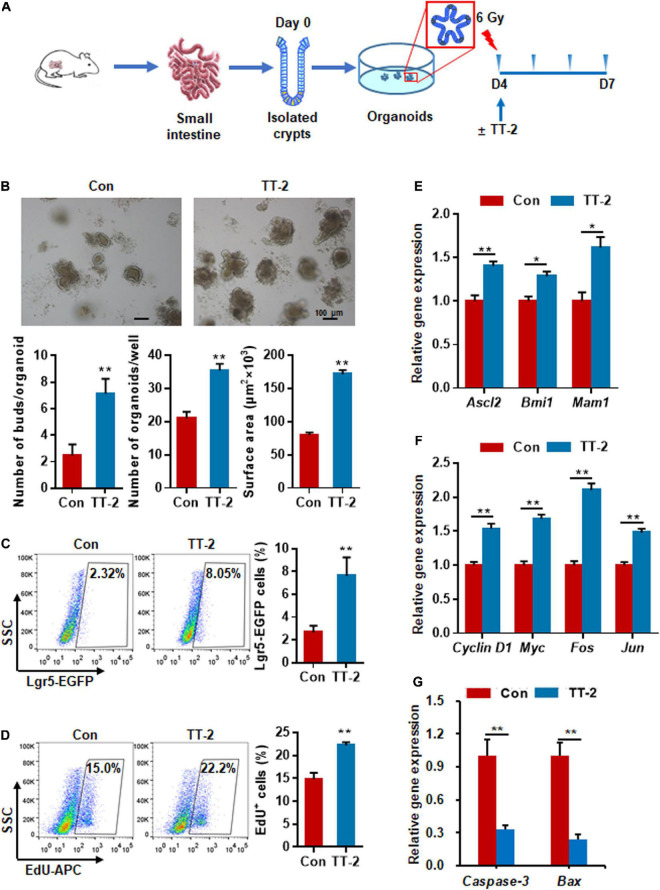
Active fraction of *Trillium tschonoskii* (TT) promoted intestinal organoid growth after irradiation. **(A)** Schematic diagram of organoid isolation and irradiation. **(B)** Representative phase contrast microscopic images of intestinal organoids cultured in presence of TT-2, and quantification of organoid numbers per well, bud numbers, and surface area of each organoid (**p < 0.01, scale bar = 100 μm). **(C)** Representative dot plots and percentages of EGFP^+^ cells in the intestinal organoids with or without TT-2. The intestinal crypts were isolated from Lgr5-EGFP-IRES-creERT2 mice (**p < 0.01). **(D)** The percentage analysis of EdU incorporation for 4 h in organoids after 6 Gy irradiation with or without the addition of TT-2 to the medium for 48 h (**p < 0.01). **(E)** qPCR for ISC-related gene expression in organoids after 6 Gy irradiation with or without TT-2 treatment for 48 h (*p < 0.05, **p < 0.01). **(F)** qPCR for proliferation-related gene expression in organoids after 6 Gy irradiation with or without TT-2 treatment for 48 h (**p < 0.01). **(G)** qPCR for apoptosis-related gene expression in organoids after 6 Gy irradiation with or without TT-2 treatment for 48 h (**p < 0.01).

To determine whether TT-2 could directly act on *in vitro* ISC proliferation, freshly isolated small intestinal crypts of normal mice were cultured for 4 days to form organoids, and then exposed to 6 Gy radiation (Figure 3A). After irradiation, cells were incubated in TT-2 containing culture medium for 3 days. The imaging data showed that TT-2 significantly increased the total organoid number, budding number, and surface area of each organoid at 0.7-, 1.9-, and 1.1-fold, respectively, compared to those in the control group on day 3 after 6 Gy irradiation (Figure 3B). By employing Lgr5-EGFP-IRES-creERT2 mice, we found that the irradiated organoids cultured with TT-2 showed a markedly increased percentage of Lgr5-EGFP^+^ cells at 1.9-fold (Figure 3C). The EdU incorporation percentage of TT-2-treated organoids was increased by 0.5-fold compared with the controls (Figure 3D). Gene expression analysis of these irradiated organoids showed that TT-2 significantly upregulated the transcription of ISC-related and proliferation-promoting genes such as *Ascl2*, *Bmi1*, *Mam1*, *Cyclin D1*, *Myc*, *Fos*, and *Jun* at 48 h after irradiation (Figures 3E,F). The apoptosis-related genes, such as *Caspase-3* and *Bax*, in the irradiated intestinal organoids were remarkably downregulated by TT-2 (Figure 3G). These observations indicated that TT-2 promoted intestinal organoid growth and ISC proliferation *in vitro*.

### *Trillium tschonoskii*-2 Enhances Repair of Intestinal Epithelium in Mice After Abdominal Irradiation

**FIGURE 4:**
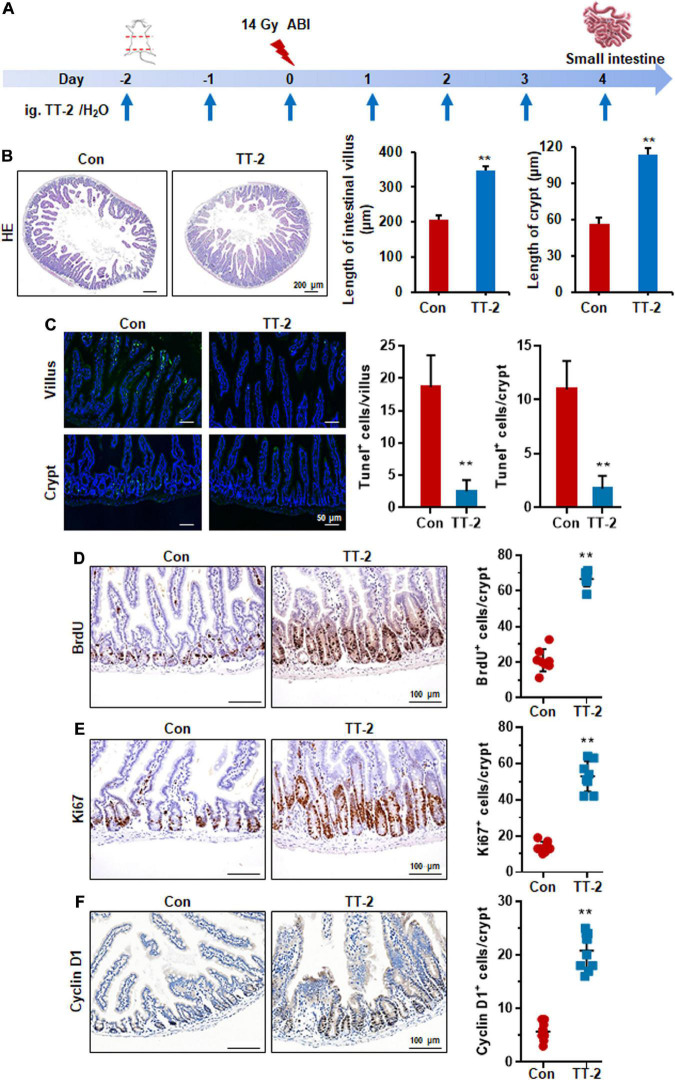
TT-2 promoted proliferation of small intestinal epithelial cells. **(A)** Time schedule for TT-2/H_2_O oral administration to mice after 14 Gy irradiation. **(B)** Representative H&E-stained sections and quantification of villus and crypt length 4 days after irradiation (**p < 0.01, scale bar = 200 μm). Three mice from each group were used. **(C)** Representative TUNEL-stained images and the quantification of the TUNEL^+^ cells in the villus and crypt of the small intestine on day 4 after 14 Gy WBI and different treatments (**p < 0.01, Scale bar = 50 μm). **(D)** Representative BrdU-stained sections and quantification of BrdU^+^ cells in crypt of small intestine after 14 Gy ABI and different treatments (**p < 0.01, scale bar = 100 μm). Three mice from each group were used. **(E)** Representative Ki67-stained sections and quantification of Ki67^+^ cells in crypt of small intestine after 14 Gy ABI and different treatments (**p < 0.01, scale bar = 100 μm). Three mice from each group were used. **(F)** Representative Cyclin D1-stained sections and quantification of Cyclin D1^+^ cells in crypt of small intestine after 14 Gy ABI and different treatments (**p < 0.01, scale bar = 100 μm). Three mice from each group were used.

**FIGURE 5:**
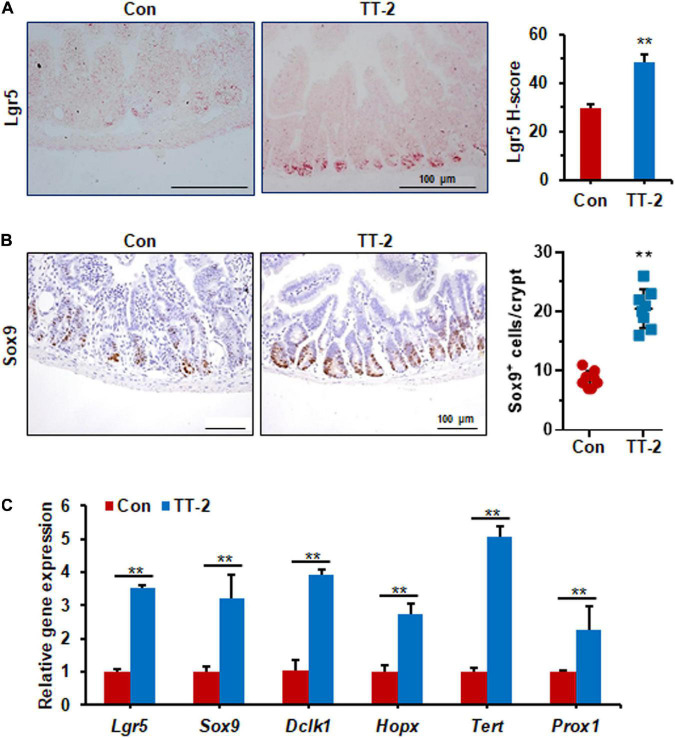
TT-2 promoted intestinal stem cell proliferation of mice after 14 Gy ABI. **(A)** Representative images of *in situ* hybridization for Lgr5 mRNA in intestinal crypts after 14 Gy ABI and different treatments (**p < 0.01, scale bar = 100 μm). Three mice from each group were used. **(B)** Representative Sox9-stained sections and quantification of Sox9^+^ cells in crypt of small intestine after 14 Gy ABI and different treatments (**p < 0.01, scale bar = 100 μm). Three mice from each group were used. **(C)** qPCR for *Lgr5, Sox9, Dclk1, Hopx, Tert*, and *Prox1* gene expression in mice after 14 Gy irradiation with or without TT-2 treatment for 96 h after ABI (**p < 0.01).

**FIGURE 6:**
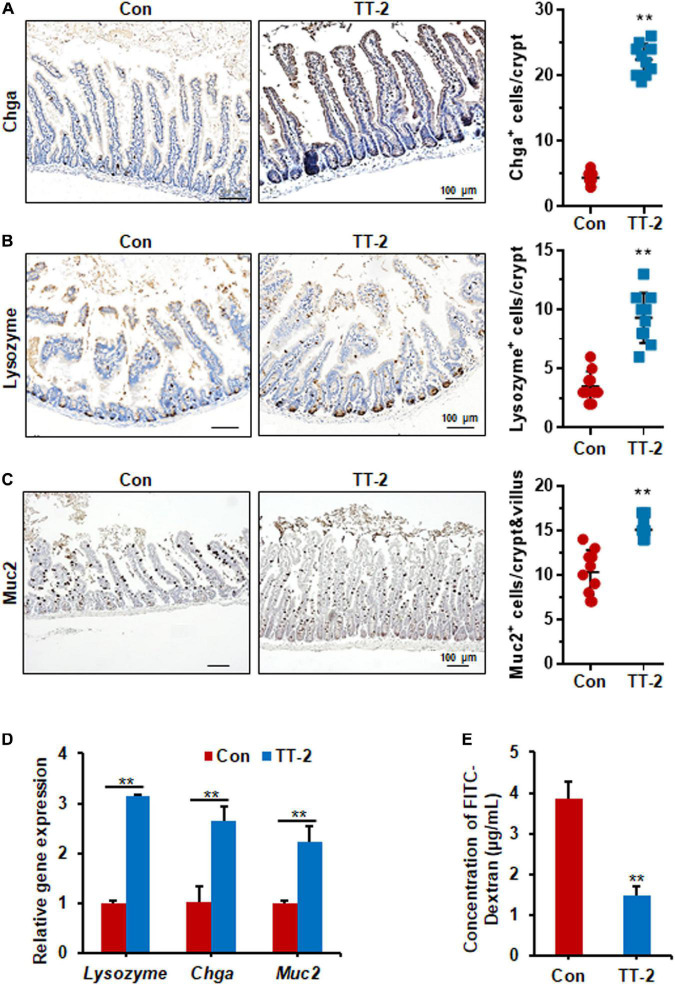
TT-2 enhanced the integrity of intestinal epithelium of mice after 14 Gy ABI. **(A)** Representative Chga-stained sections and quantification of Chga^+^ cells in crypt of small intestine after 14 Gy ABI and different treatments (**p < 0.01, scale bar = 100 μm). Three mice from each group were used. **(B)** Representative lysozyme-stained sections and quantification of lysozyme^+^ cells in crypt of small intestine after 14 Gy ABI and different treatments (**p < 0.01, scale bar = 100 μm). Three mice from each group were used. **(C)** Representative Muc2-stained sections and quantification of Muc2^+^ cells in crypt of small intestine after 14 Gy ABI and different treatments (**p < 0.01, scale bar = 100 μm). Three mice from each group were used. **(D)** qPCR for differentiation-related gene expression in mouse intestinal epithelial cells after 10 Gy irradiation with or without TT-2 treatment for 48 h (**p < 0.01). **(E)** The FITC-Dextran level in the serum of mice on day 4 after 14 Gy ABI and treatment with water with or without TT-2 from day −2 to 4 (**p < 0.01).

Acute and lethal intestinal crypt injury is triggered upon exposure to high doses of radiation (>12 Gy) (Kuhnert et al., 2004; Chen et al., 2014). To evaluate the beneficial effects of TT-2 on radiation-induced intestinal tissue damage, C57BL/6 mice were exposed to 14 Gy ABI on day 0, and were gavaged TT-2 or H_2_O every day from day −2–4 (Figure 4A). It has been reported that ISCs in the crypt base are initially inactive, are activated within several hours to 4 days after irradiation, and are mainly responsible for crypt regeneration (Booth et al., 2012; Yan et al., 2012). Therefore, we collected intestinal tissue 4 days after ABI. Notably, oral administration of TT-2 improved intestinal tissue morphology (Figure 4B). Histological analysis of H&E-stained intestinal sections showed that TT-2 administration significantly increased intestinal villus and crypt lengths compared to those in the control group (Figure 4B). The villus and crypt length in the TT-2 group was remarkably higher than in the control group, 4 days after irradiation (Figure 4B). After treatment with TT-2, the number of apoptotic cells in the villus and crypt was significantly decreased (Figure 4C). To gain insight into the proliferative status of the intestinal crypts upon irradiation and TT-2 treatment, we performed a 12 h bromodeoxyuridine (BrdU) tracer experiment. We observed a 2.1-fold increase in the percentage of BrdU^+^ cells in the small intestine section of TT-2-treated mice than that in the controls 4 days after irradiation (Figure 4D). The immunohistochemical staining results demonstrated significant increases in the positive cell number of Ki67 and Cyclin D1 in the crypt of TT-2-treated mice, as shown by the brown staining of the intestinal tissue sections (Figures 4E,F). On day 4 post ABI, TT-2- treated intestinal tissue sections showed significant increases in the numbers of Ki67^+^ cells and Cyclin D1^+^ cells by 2.8-fold and 1.1-fold, respectively (Figures 4E,F). We also detected the expression of Lgr5 mRNA and Sox9 protein in the small intestine sections, these are active ISC markers and are critical for crypt regeneration. Using an *in situ* hybridization assay, we found that TT-2 administration significantly improved the expression of Lgr5 mRNA in the intestinal crypts (Figure 5A). We also found that TT-2 administration significantly enhanced the percentage of Sox9^+^ cells compared with that in the control group, 4 days after ABI (Figure 5B). The expression levels of the ISC-related genes, such as *Lgr5, Sox9, Dclk1, Hopx, Tert*, and *Prox1*, significantly increased after TT-2 treatment at 96 h after ABI (Figure 5C). To further evaluate the role of TT-2 in repairing intestinal epithelial cells after irradiation, we analyzed several types of intestinal epithelial cells after 14 Gy ABI, including enteroendocrine, Paneth, and goblet cells. Immunohistochemical staining results showed that TT-2 administration significantly increased the positivity rate of Chga for enteroendocrine cells, Lysozyme for Paneth cells, and Muc2 for goblet cells in the intestinal sections (Figures 6A–C). Gene expression analysis of the irradiated mice showed that TT-2 significantly upregulated the expression of *Chga*, *Lysozyme*, and *Muc2* genes at 48 h after irradiation (Figure 6D). We then employed FITC-Dextran assay to evaluate the effect of TT-2 on intestinal permeability in mice after irradiation. TT-2 administration significantly reduced the FITC-Dextran level in the serum of mice at day 4 after 14 Gy irradiation (Figure 6E). The result indicated that TT-2 administration decreased intestinal epithelial permeability and prevented gut leakiness to a greater extent in mice after irradiation. These data suggested that TT-2 promoted ISC differentiation and the repair of the intestinal epithelium of mice after high dosage of ABI.

## Discussion

In this study, we discovered a novel function of an active fraction of TT, TT-2, it promoted intestinal epithelial repair after irradiation. To the best of our knowledge, this is the first report on the mitigative function of this natural product on radiation-induced intestinal toxicity. TT-2 enhanced colony formation, promoted cell proliferation, and inhibited apoptosis of the irradiated intestinal crypt cell line, IEC-6. TT-2 also enhanced the growth of an irradiated intestinal organoid model. Results of *in vivo* experiments indicated that oral administration of TT-2 significantly improved the expression of ISC-related genes and enhanced crypt regeneration and epithelial repair of radiation-damaged intestinal tissue.

Radiotherapy is often used to kill residual tumor cells in clinics. However, abdominal or pelvic tumor radiotherapy is painful for patients with various symptoms, such as AGS, which limits the dosage of radiation. The lack of effective drugs with mitigative effects on AGS has stimulated efforts to find new and potential drugs to reduce radiation-induced intestinal damage and enhance intestinal epithelial repair. Primary screening of effective agents using intestinal cell lines or organoid models combined with special phenotype analysis will provide a clue to discover natural products or compounds with radiation countermeasure potential. China has abundant traditional herbs and several natural herbal products have been reported to play a beneficial role in tissue-injury repair (Meng et al., 2020). Hence, we established a screening system based on counting the numbers of colonies formed by irradiated IEC-6 cells treated with different constituents from a TCM library, which contains different extracts of TT. Some scientists are devoting to isolate and purify the chemical ingredients of TT and investigate the function of them. Accumulated evidence indicates that there are various constituents in TT with diverse function (Yan et al., 2016; Wang et al., 2018; Wang B. et al., 2020). The total saponins of TT showed the protective and repair-promoting effects on injured brain cells (Ludwig et al., 2018). However, a kind of chemical compounds, spirostanol saponins, showed an inhibitory role on the growth or/and metastasis of tumor cells (Huang et al., 2011). Interestingly, we found that a number of furostanol saponins isolated from TT promoted the expansion of hematopoietic stem and progenitor cells (Wang B. et al., 2020). Here, we also tested the effect of these saponin ingredients from TT on irradiated intestinal cell proliferation in primary screening step. TT-4 to TT-6, which mainly consist of steroidal saponins, showed less effect on enhancing the colony number of irradiated IEC-6 cells than TT-2 (data not shown). Thus, we further investigated the role of TT-2 on irradiated intestinal cells by using other proliferation-related detection criteria. The ingredients of TT-2 are different from TT-4 to TT-6, which mainly consists of oligosaccharide components according to our purification method and sample analysis results. It is difficult to further purify each oligosaccharide from TT-2 due to the limited oligosaccharide purification technique (Moravcova et al., 2021). We first found the novel function of TT-2 in improving irradiated intestinal epithelial regeneration by employing the colony-forming screening model. EdU incorporation detection and cell apoptosis analysis results indicated that TT-2 enhanced regeneration mainly by increasing cell proliferation and suppressing the apoptosis of irradiated IEC-6 cells. Results of gene expression analysis further supported these observations, indicating that TT-2 has a positive effect on the survival and proliferation of irradiated IEC-6 cells. We also confirmed the similar effect of TT-2 on human intestinal epithelial cell line HIEC-6. TT-2 significantly promoted the incorporation of EdU in the nucleus, increased the expression of proliferation-related genes and inhibited the expression of apoptosis-related genes in irradiated HIEC-6 (Supplementary Figures 1A–C). Notably, TT-2 showed no significant cytotoxic effect on unirradiated intestinal epithelial cells, and there was no obvious difference in the colony-forming number of unirradiated IEC-6 cells treated with different concentration of TT-2 (Supplementary Figures 2A,B).

To reduce the cost of research and development of new drugs and the risk of failure, many labs and pharmaceutical companies have focused on the development of predictive and reliable cell or tissue models for primary screening of drug-candidate efficacy *in vitro*. In recent years, several breakthroughs have been achieved regarding the culture of ISC-derived intestinal organoids (Ootani et al., 2009; Sato et al., 2009; Jung et al., 2011; Mahe et al., 2013; Wang et al., 2013). Intestinal organoids have the epithelial architecture and physiological characteristics of the intestine, and are more effective and useful models for investigating factors that regulate ISC self-renewal, proliferation, and differentiation (Sato et al., 2011). Patient-derived organoids have proved to be a reliable model to test the treatment response of metastatic gastrointestinal cancer (Vlachogiannis et al., 2018). Here, we established an irradiated small intestinal organoid model to test the efficacy of primary-screened special active fractions of natural products. Utilizing the radiation-injured intestinal organoid model, we found that TT-2 significantly increased the total organoid numbers and budding rate of irradiated intestinal organoids, most likely by increasing proliferation-related gene expression and decreasing apoptosis-related gene expression in the irradiated organoids. These data provide compelling evidence for a novel role of TT-2 in promoting intestinal organoid growth after *in vitro* irradiation. TT-2 addition to the culture medium showed no significant effect on the growth of unirradiated intestinal organoids (Supplementary Figures 3A,B). There was no significant difference in the expression levels of the ISC-related genes, proliferation-related genes, and anti-apoptosis-related genes between Con- and TT-2-treated unirradiated organoids (Supplementary Figures 3C–E). These results indicated that TT-2 had no significant proliferation-enhancing role on normal intestinal epithelium. The beneficial influence of TT-2 on irradiated intestinal organoids prompted us to perform animal experiments to evaluate the effect of TT-2 on radiation-induced intestinal damage. We demonstrated that TT-2 is a potent radiation countermeasure, evidenced by the fact that oral administration of TT-2 into mice after lethal doses of ABI significantly increased the length of the intestinal crypts and villi by promoting intestinal cell and ISC proliferation. Several studies indicate that *in vivo* function of chemicals or biomaterials is complex and might involve in indirectly regulatory manner on tissue repairing (Bressan et al., 2019; Peng et al., 2020). Given that intestinal tissue contains multiple cell types and cytokines, it needs to do more work to investigate whether TT-2 can indirectly regulate intestinal cell regeneration *via* other type of cells or co-factors. In recent years, mesenchymal stem cells (MSCs) or their exosomes have demonstrated enhancing-repair function in injured tissue (Ballini et al., 2017; Soontararak et al., 2018). It is valuable to further evaluate the combination effect of MSC transplantation and TT-2 administration on radiation-injured animal model in the future.

## Conclusion

Our data demonstrate a novel role for an active fraction of *Trillium tschonoskii*, TT-2, it enhanced the colony forming numbers and improved the proliferation of irradiated intestinal crypt cells. More importantly, TT-2 significantly accelerated intestinal organoid growth and increased Lgr5^+^ ISC numbers after radiation exposure. TT-2 plays a beneficial role in irradiated intestinal crypt cells and organoids mainly by promoting the expression of proliferation-related genes and inhibiting the expression of apoptosis-related genes. *In vivo* animal experiments showed that TT-2 remarkably enhanced intestinal crypt cell proliferation and new crypt regeneration after irradiation damage. Notably, the administration of TT-2 significantly decreased intestinal epithelial permeability and reduced gut leakiness in irradiated mice. Overall, our data revealed that this active fraction of TT has the potential to be further developed for use in clinics to treat patients with AGS by enhancing intestinal epithelium repair.

## Data Availability Statement

The original contributions presented in the study are included in the article/Supplementary Material, further inquiries can be directed to the corresponding author/s.

## Ethics Statement

The animal study was reviewed and approved by the Institutional Animal Care and Use Committee of Laboratory Animal Center.

## Author Contributions

YL: conception and design, data analysis and interpretation, manuscript writing, and revising. XTP and BM: data analysis and interpretation. FS and SW: collection and assembly of data, and manuscript writing. XP, JZ, and XC: separation and extraction of fractions of Traditional Chinese Medicine. ZF and LH: helping in conducting *in vitro* cellular and molecular biology experiments. All authors contributed to the article and approved the submitted version.

## Conflict of Interest

The authors declare that the research was conducted in the absence of any commercial or financial relationships that could be construed as a potential conflict of interest.

## Publisher’s Note

All claims expressed in this article are solely those of the authors and do not necessarily represent those of their affiliated organizations, or those of the publisher, the editors and the reviewers. Any product that may be evaluated in this article, or claim that may be made by its manufacturer, is not guaranteed or endorsed by the publisher.
