# Supplementary material for: An Active Fraction of Trillium tschonoskii Promotes the Regeneration of Intestinal Epithelial Cells After Irradiation
Source: Front Cell Dev Biol. 2021 Nov 2;9:745412. doi: 10.3389/fcell.2021.745412 (PMC8593212; doi:10.3389/fcell.2021.745412)
Supplement: Supplementary file 1 [file Table_1.docx]

**Supplementary Table 1 Primers for qPCR**

| Genes | Forward primer (5’-3’) | Reverse primer (5’-3’) |
| --- | --- | --- |
| *GAPDH(R)* | GGCATGGACTGTGGTCATGAG | TGCACCACCAACTGCTTAGC |
| *Cyclin D1(R)* | GGAGCCCCTGAAGAAGAG | CCAGAAGCAGTTCCATTTG |
| *Myc(R)* | GAAGAACAAGATGATGAGGAA | GCTGGTGAGTAGAGACAT |
| *Bcl2(R)* | ACTGAGTACCTGAACCGGCATC | GGAGAAATCAAACAGAGGTCGC |
| *P53(R)* | TCGAGATGTTCCGAGAGCTGAATG | CTTCTTGGTCTTCGGGTAGCTG |
| *HPRT(M)* | TCAGTCAACGGGGGACATAAA | GGGGCTGTACTGCTTAACCAG |
| *Cyclin D1(M)* | CTGTGCTGCGAAGTGGAAACCAT | TTCATGGCCAGCGGGAAGACCTC |
| *Myc(M)* | CCCTATTTCATCTGCGACGAG | GAGAAGGACGTAGCGACCG |
| *Fos(M)* | CCAGTCAAGAGCATCAGCAA | AAGTAGTGCAGCCCGGAGTA |
| *Jun(M)*  *Bmi1(M)*  *Ascl2(M)*  *Mam1(M)*  *Caspase-3(M)*  *Bax(M)* | ACTCGGACCTTCTCACGTC  AAATCCCCACTTAATGTGTGTCC  AAGCACACCTTGACTGGTACG  GCCCGACGAAGACATGAAGG  CTCGCTCTGGTACGGATGTG  AGACAGGGGCCTTTTTGCTAC | GGTCGGTGTAGTGGTGATGT  CTTGCTGGTCTCCAAGTAACG  AAGTGGACGTTTGCACCTTCA  CTTGTGGAGAACCTAACTGCTC  TCCCATAAATGACCCCTTCATCA  AATTCGCCGGAGACACTCG |
| *Dclk1(M)* | CTGGGTTAATGATGATGGTCTCC | TCCTGGTTGTTGGTAGTAGTCC |
| *Tert(M)* | CCAACACTGTTATTGAGACCCTG | GGCCTGTAACTAGCGGACAC |
| *Prox1(M)* | GTGGTGCAACACGCAGATG | TGCCACCGTTTTTGTTCATGT |
| *Lgr5(M)* | GGACCAGATGCGATACCGC | CAGAGGCGATGTAGGAGACTG |
| *Sox9(M)* | CGGAACAGACTCACATCTCTCC | GCTTGCACGTCGGTTTTGG |
| *Chga(M)* | CACGGGAGACAGTGAGAGAG | TCCGACTGACCATCATCTTTCT |
| *Lysozyme(M)* | GAGACCGAAGCACCGACTATG | CGGTTTTGACATTGTGTTCGC |
| *Muc2(M)* | GCTGACGAGTGGTTGGTGAATG | GATGAGGTGGCAGACAGGAGAC |
| *Hopx(M)* | TCTCCATCCTTAGTCAGACGC | GGGTGCTTGTTGACCTTGTT |
| *GAPDH(H)* | GGCATGGACTGTGGTCATGAG | TGCACCACCAACTGCTTAGC |
| *Bcl-2(H)* | ACTGAGTACCTGAACCGGCATC | GGAGAAATCAAACAGAGGTCGC |
| *Cyclin D1(H)* | GGAGCCCCTGAAGAAGAG | CCAGAAGCAGTTCCATTTG |
| *Myc(H)* | GAAGAACAAGATGATGAGGAA | GCTGGTGAGTAGAGACAT |
| *p53(H)* | GAGGTTGGCTCTGACTGTACC | TCCGTCCCAGTAGATTACCAC |
| *Caspase-3(H)* | GAAATTGTGGAATTGATGCGTGA | CTACAACGATCCCCTCTGAAAAA |
| *Fos(H)* | CACTCCAAGCGGAGACAGAC | AGGTCATCAGGGATCTTGCAG |
| *Jun(H)* | TCCAAGTGCCGAAAAAGGAAG | CGAGTTCTGAGCTTTCAAGGT |
