# Supplementary material for: An Active Fraction of Trillium tschonoskii Promotes the Regeneration of Intestinal Epithelial Cells After Irradiation
Source: Front Cell Dev Biol. 2021 Nov 2;9:745412. doi: 10.3389/fcell.2021.745412 (PMC8593212; doi:10.3389/fcell.2021.745412)

Fig. 1 B Representative colony image formed by IEC-6 cells after 10 Gy irradiation and addition of DMSO, or TT-2 to the culture medium

Con

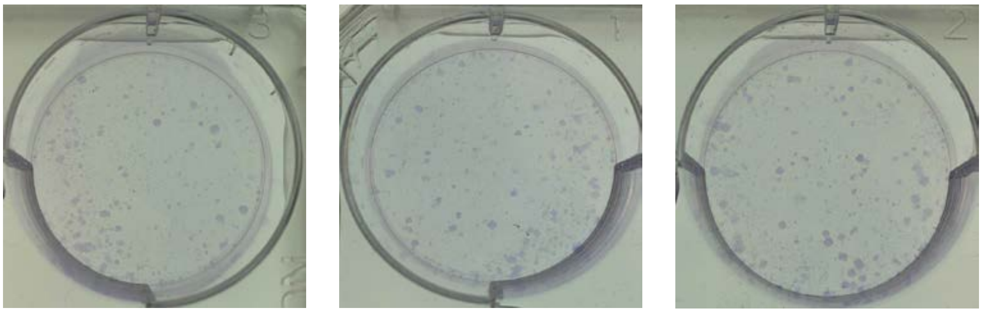

TT-2

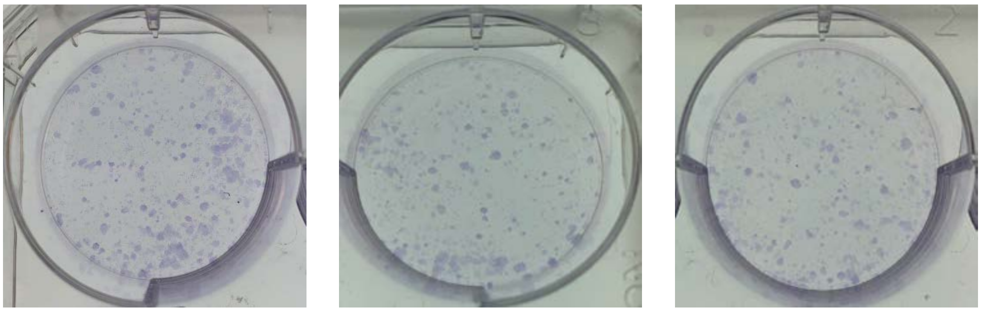

Fig. 1 C Representative colony staining image formed by IEC-6 cells after 10 Gy irradiation and addition of TT-2 or samples isolated after elution with different concentrations of ethanol (TT-2-0, TT-2-15, and TT-2-50)

Con

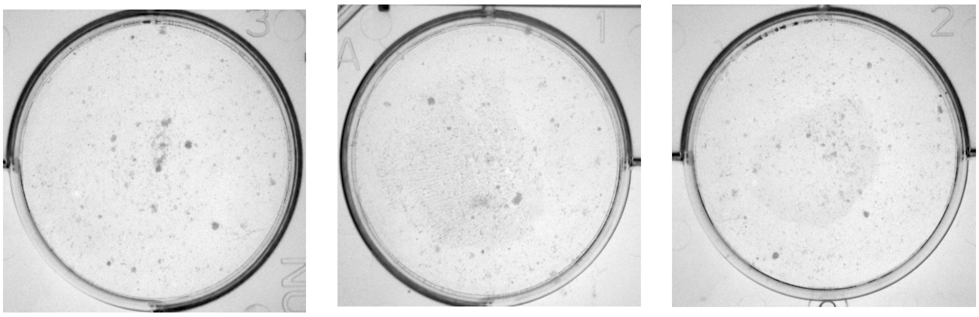

TT-2

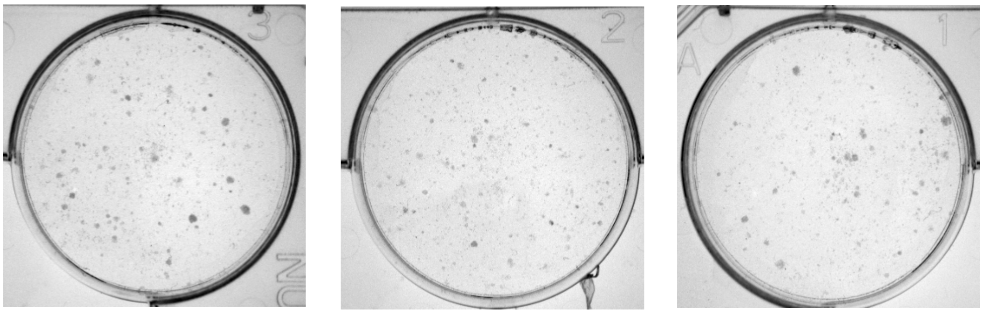

TT-2-0

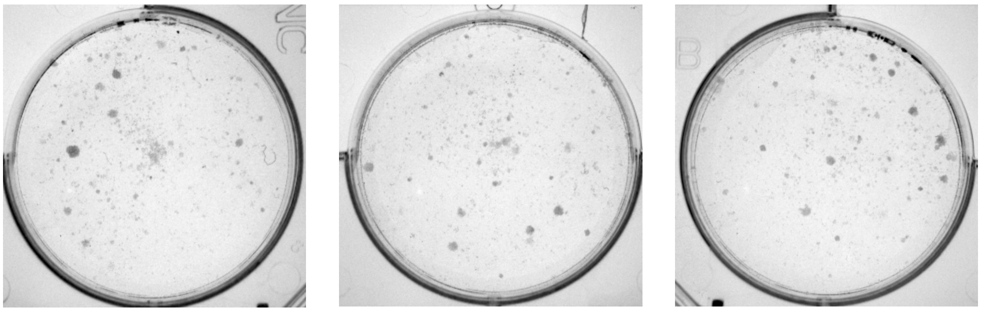

TT-2-15

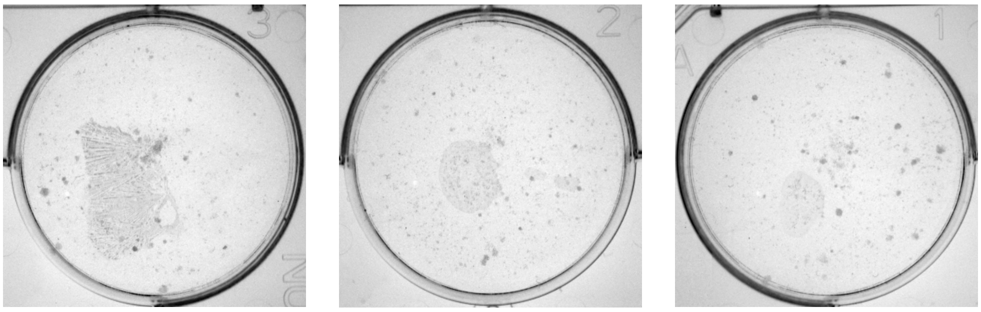

TT-2-50

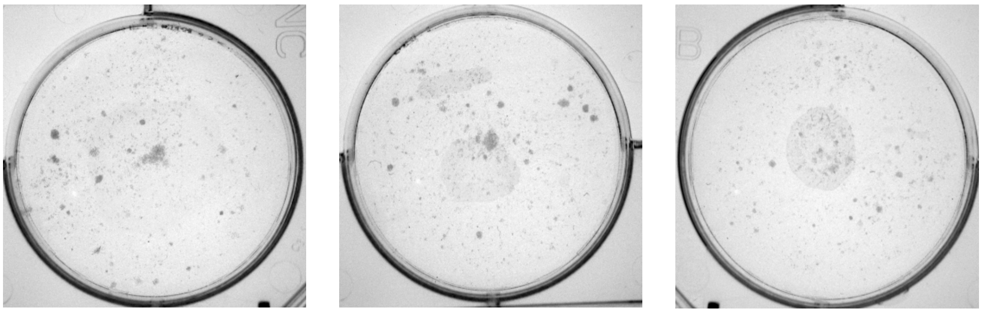

Fig. 2 A Immunostaining analysis of EdU in IEC-6 cells after irradiation with or without TT-2 or TT-2-0 for 48 h (scale bar = 80 μm).

Con

EdU DAPI Merge

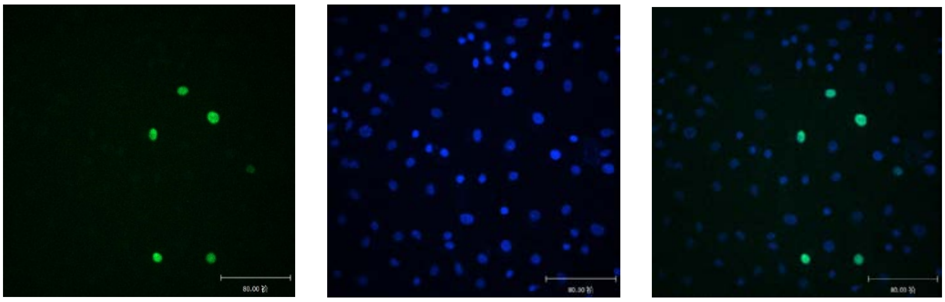

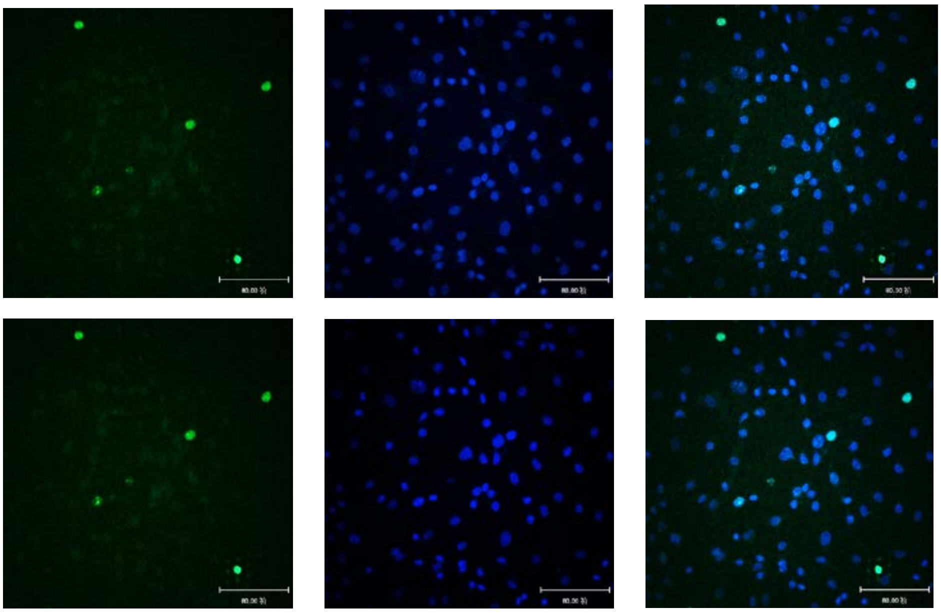

TT-2

EdU DAPI Merge

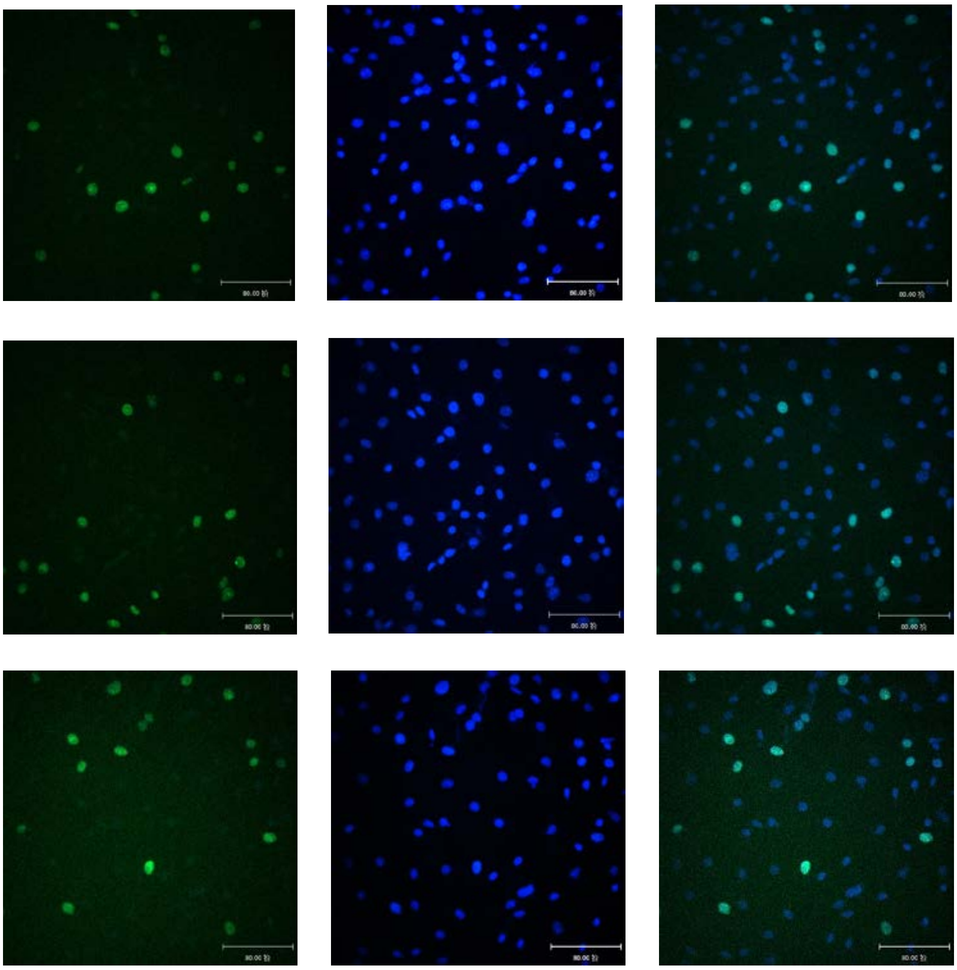

TT-2-0

EdU DAPI Merge

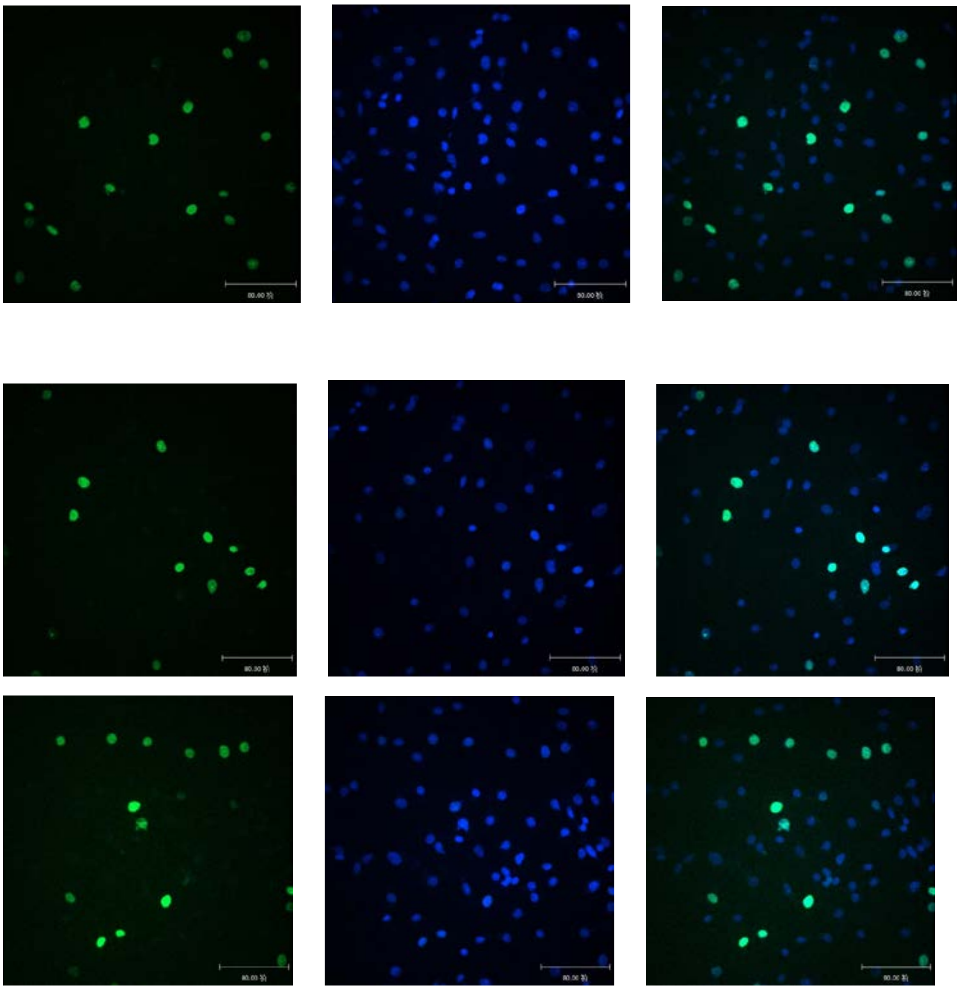

Fig. 3 B Representative phase contrast microscopic images of intestinal organoids cultured in presence of TT-2 (scale bar = 100 μm).

Con
10×

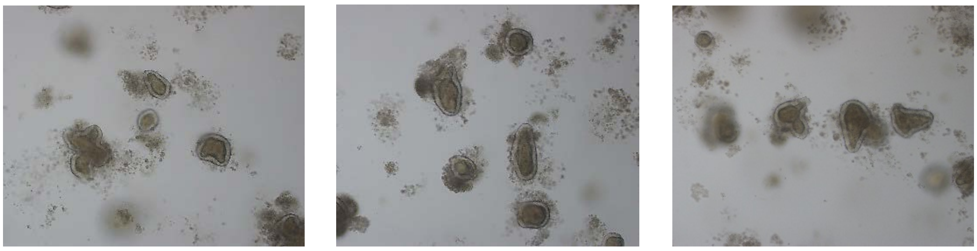

TT-2
10×

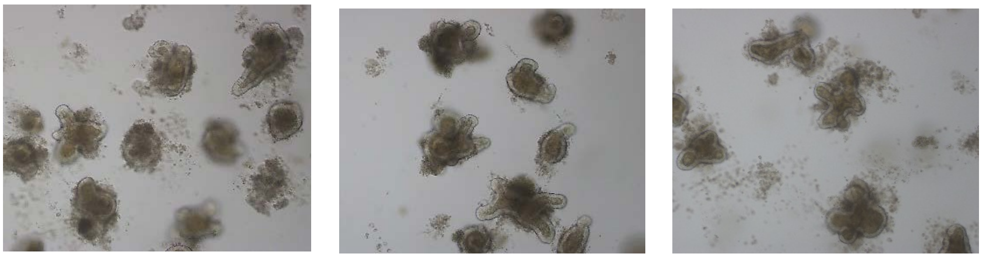

Fig. 4 B Representative H&E-stained sections of small intestine four days after irradiation (scale bar = 200 μm).

Con

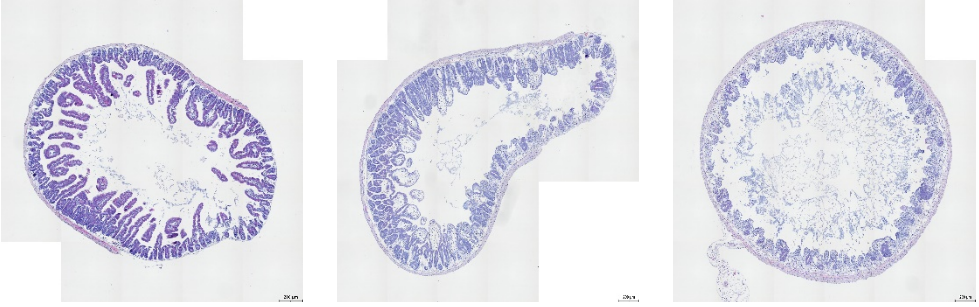

TT-2

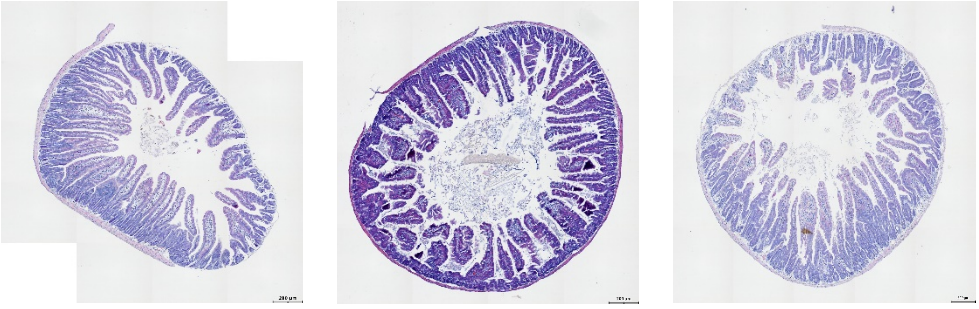

Fig. 4 C Representative Tunel-stained images of the Tunel^{+} cells in the villus and crypt of the small intestine on day 4 after 14 Gy WBI and different treatments (scale bar = 50 μm).

Con

Tunel DAPI Merge

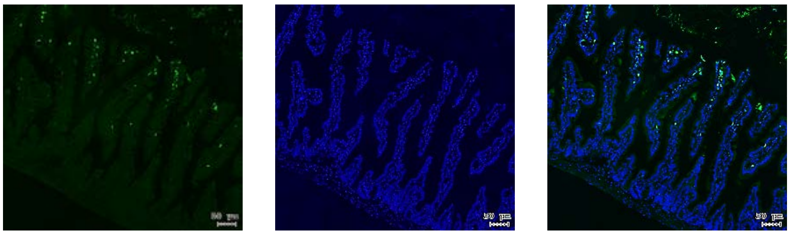

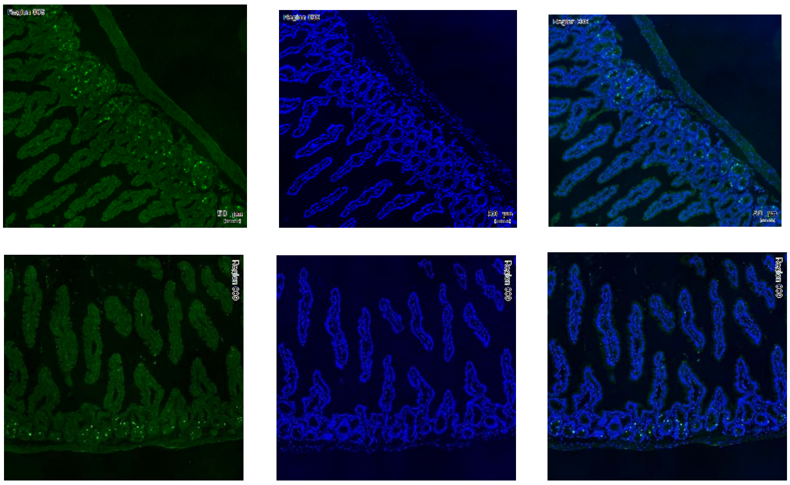

TT-2

Tunel DAPI Merge

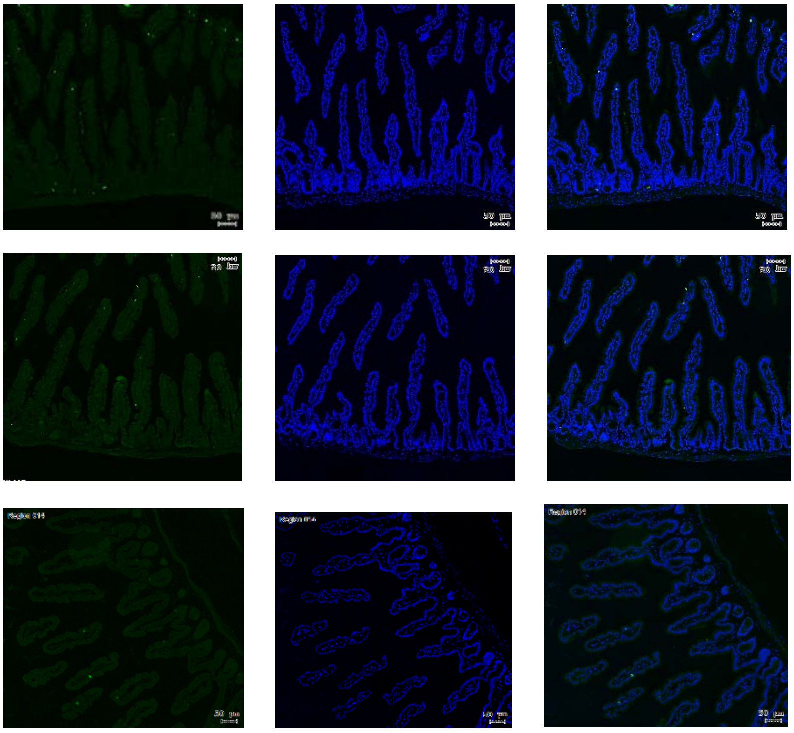

Fig. 4 D Representative BrdU-stained sections of small intestine after 14 Gy ABI and different treatments (scale bar = 100 μm).

Con

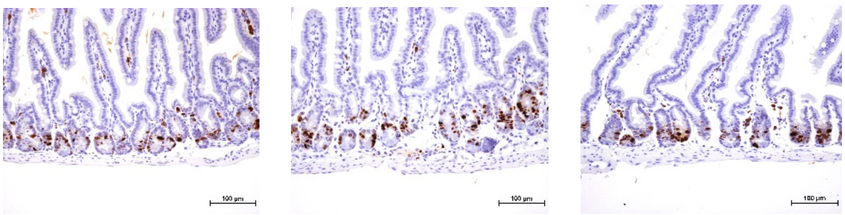

TT-2

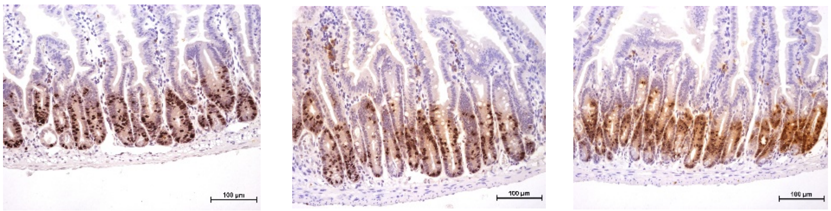

Fig. 4 E Representative Ki67-stained sections of small intestine after 14 Gy ABI and different treatments (scale bar = 100 μm).

Con

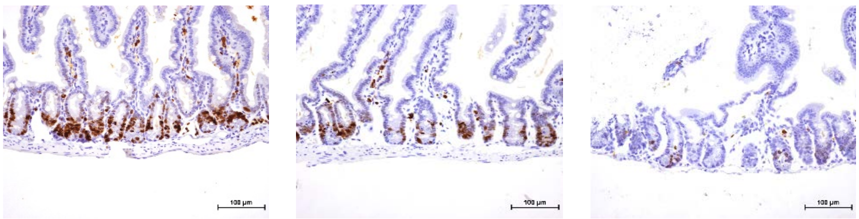

TT-2

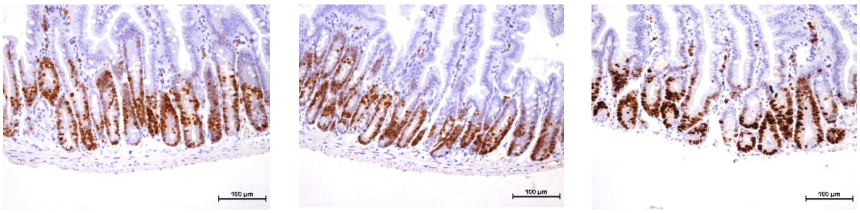

Fig. 4 F Representative Cyclin D1-stained sections of small intestine after 14 Gy ABI and different treatments (scale bar = 100 μm).

Con

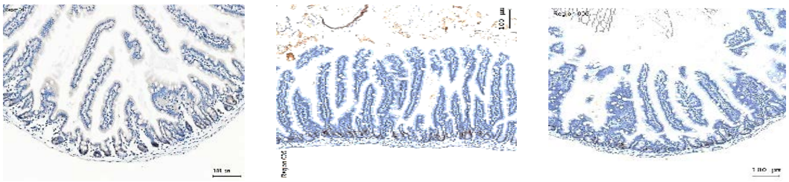

TT-2

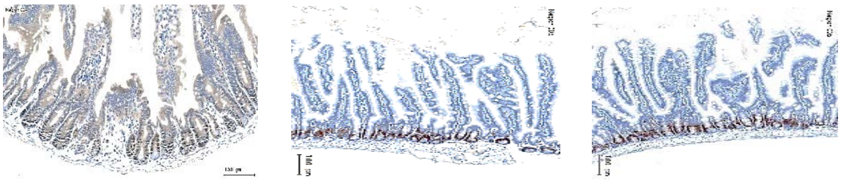

Fig. 5 A Representative images of in situ hybridization for Lgr5 mRNA in intestinal crypts after 14 Gy ABI and different treatments (scale bar = 100 μm).

Con

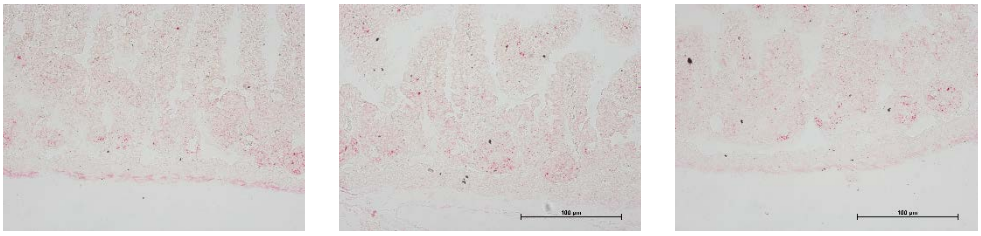

TT-2

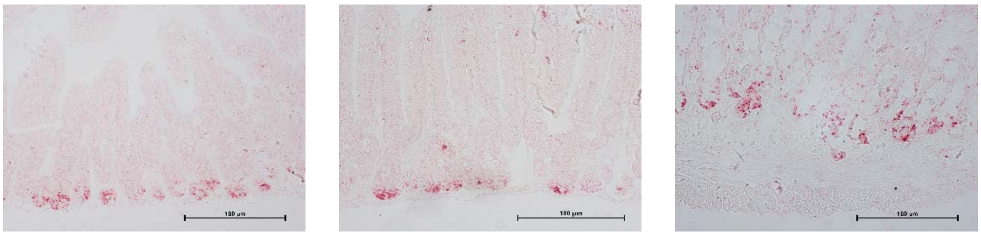

Fig. 5 B Representative Sox9-stained sections of small intestine after 14 Gy ABI and different treatments (scale bar = 100 μm).

Con

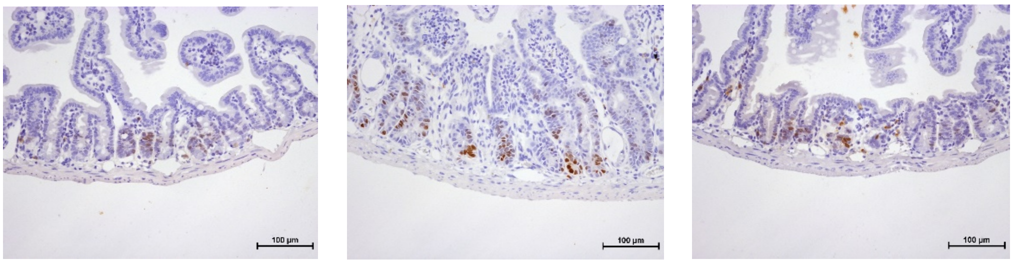

TT-2

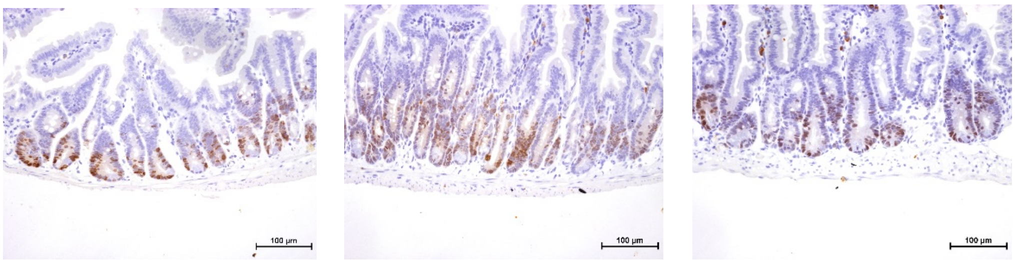

Fig. 6 A Representative Chga-stained sections of small intestine after 14 Gy ABI and different treatments (scale bar = 100 μm).

Con

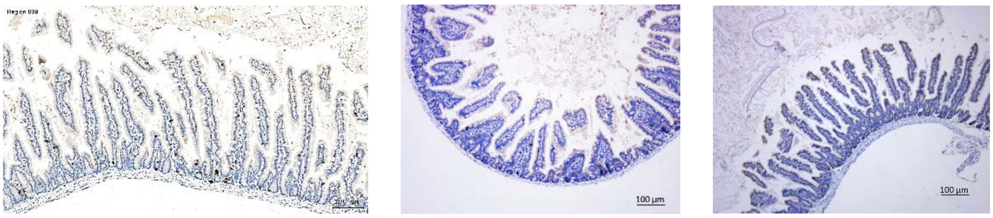

TT-2

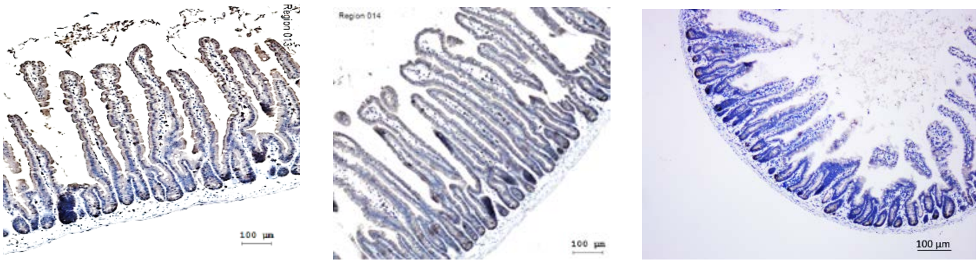

Fig. 6 B Representative lysozyme-stained sections of small intestine after 14 Gy ABI and different treatments (scale bar = 100 μm).

Con

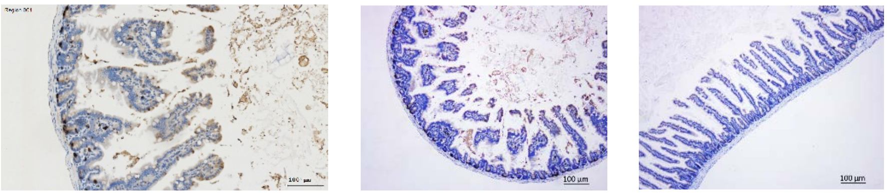

TT-2

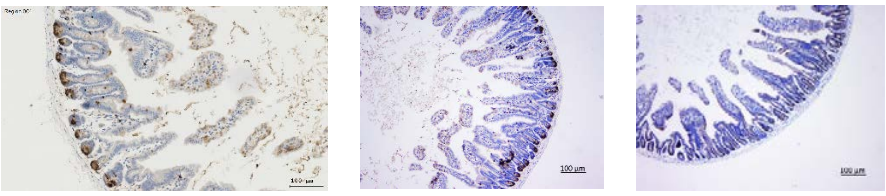

Fig. 6 C Representative Muc2-stained sections of small intestine after 14 Gy ABI and different treatments (scale bar = 100 μm).

Con

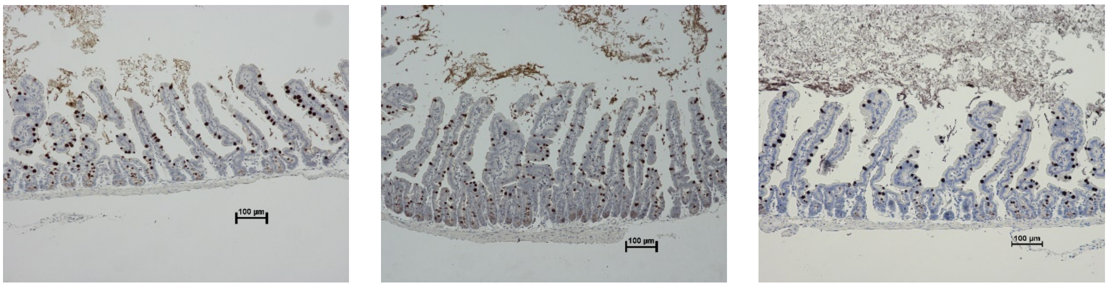

TT-2

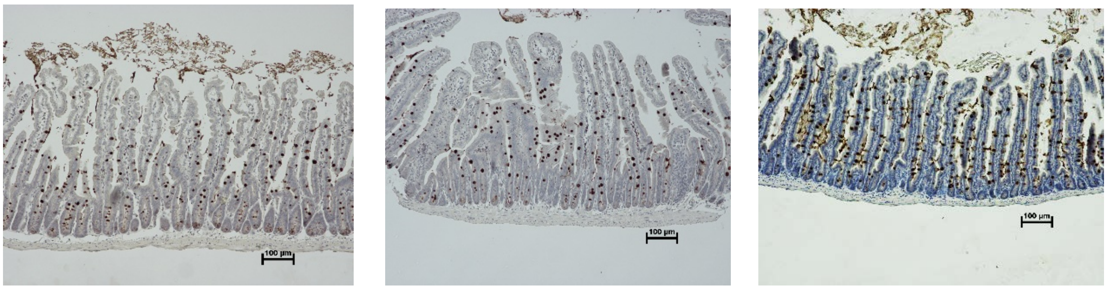

Fig. S2 B Representative colony image formed by IEC-6 cells after adding different concentrations of TT-2 to the culture medium

TT-2 0 μg/mL (Con)

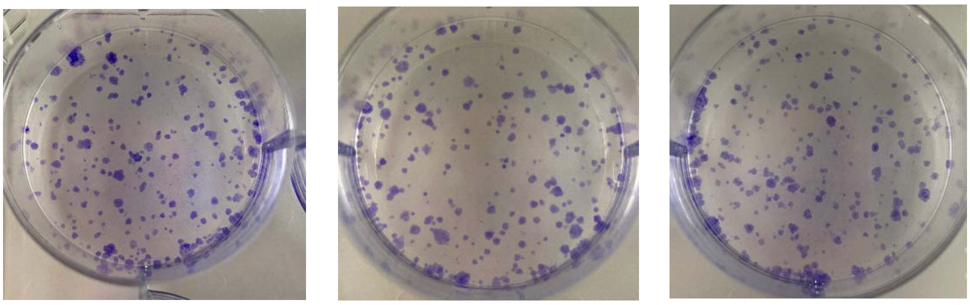

TT-2 2 μg/mL

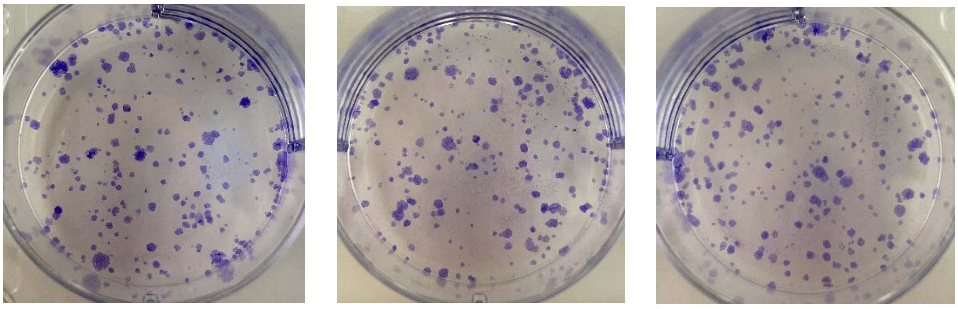

TT-2 5 μg/mL

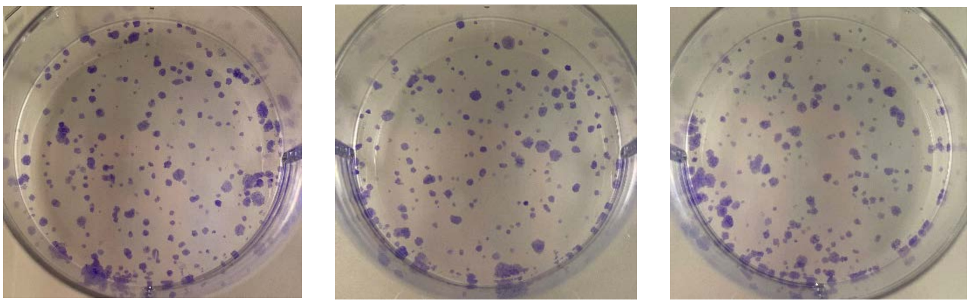

TT-2 10 μg/mL

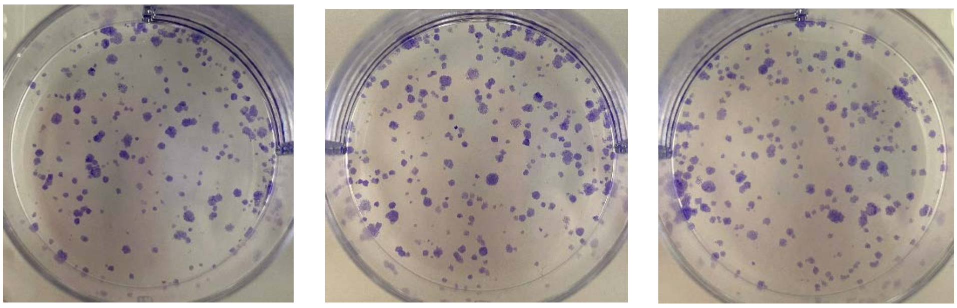

TT-2 20 μg/mL

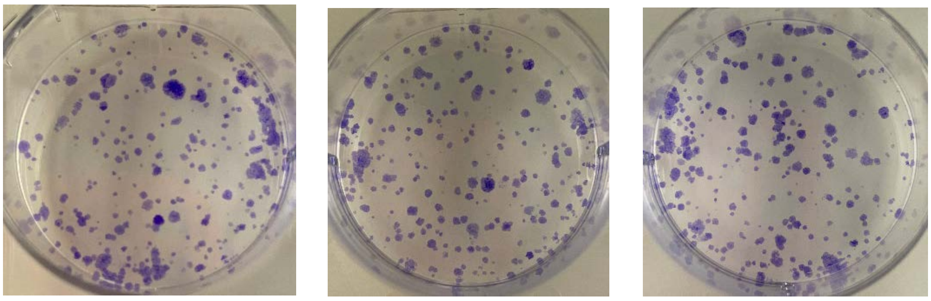

TT-2 50 μg/mL

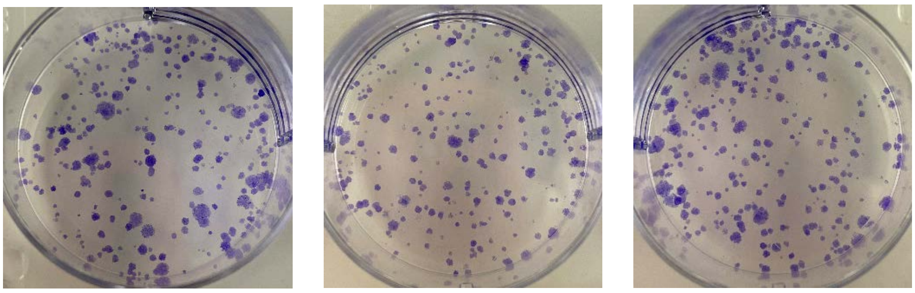

Fig. S3 B Representative phase contrast microscopic images of intestinal organoids cultured in presence of TT-2 (scale bar = 100 μm).

Con

10×

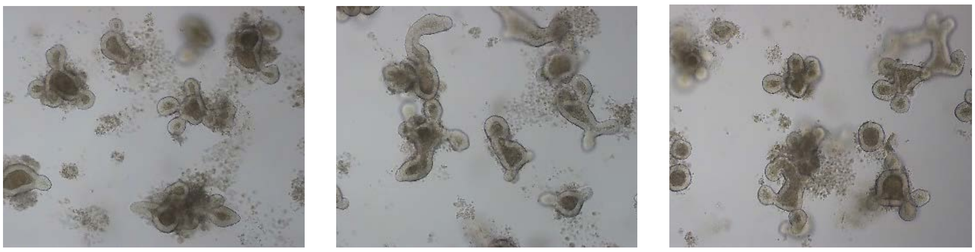

TT-2

10×

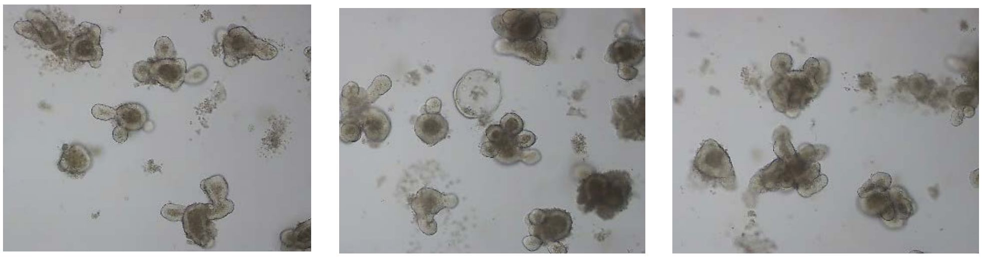

Supplement: Supplementary file 2 [file Data_Sheet_1.ZIP › Raw data/Raw microscopy images.pdf]
