# Supplementary material for: An Active Fraction of Trillium tschonoskii Promotes the Regeneration of Intestinal Epithelial Cells After Irradiation
Source: Front Cell Dev Biol. 2021 Nov 2;9:745412. doi: 10.3389/fcell.2021.745412 (PMC8593212; doi:10.3389/fcell.2021.745412)
Supplement: Supplementary file 3 [file Data_Sheet_2.docx]

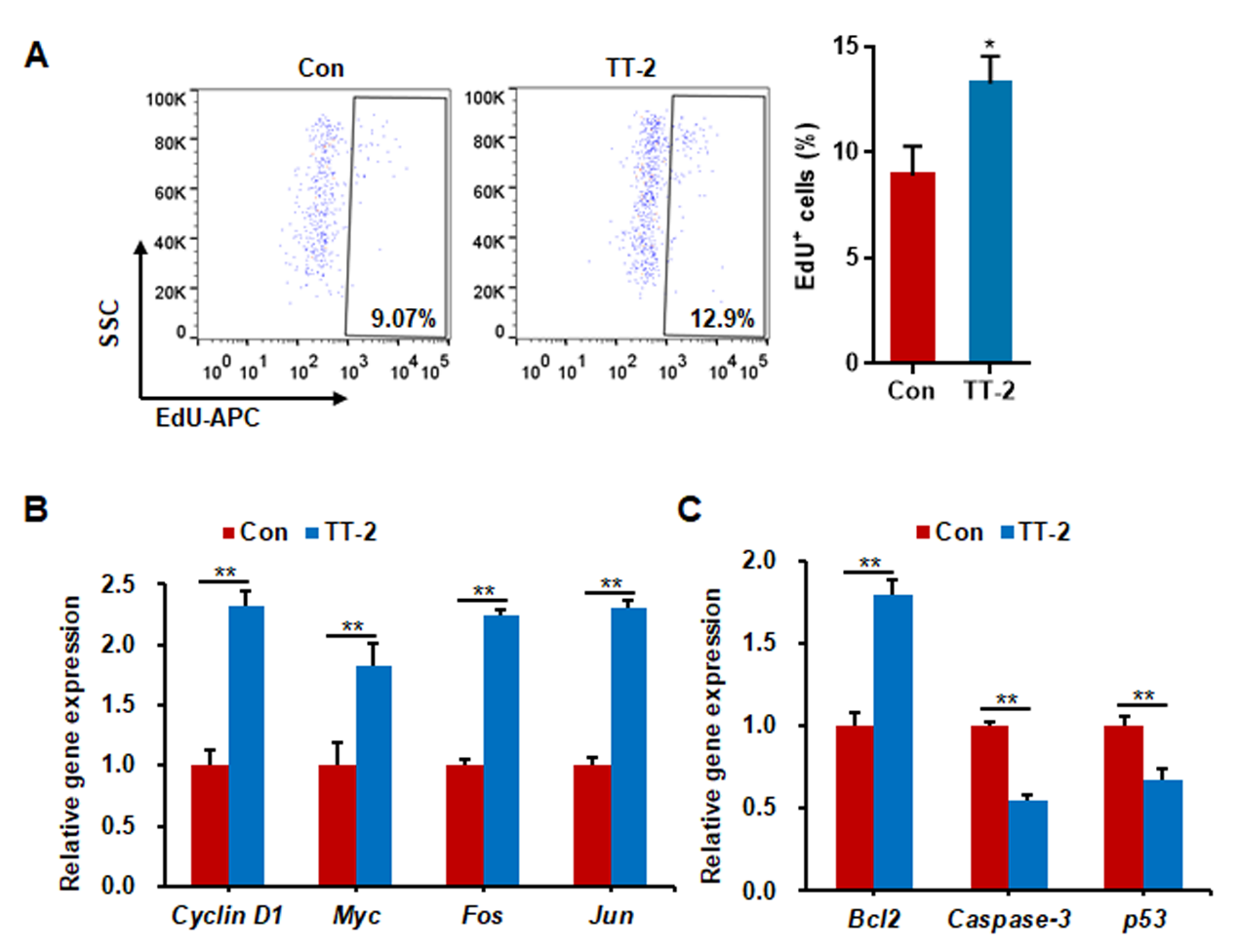
Supplementary Figure 1. Active fraction of *Trillium tschonoskii* (TT) promoted proliferation and inhibited apoptosis of HIEC-6 cells after irradiation.

1. Percentage analysis of EdU incorporation for 4 h in HIEC-6 cells after 10 Gy irradiation with or without TT-2 (*p < 0.05).
2. qPCR for proliferation-related gene expression in HIEC-6 cells after 10 Gy irradiation with or without TT-2 treatment for 48 h (**p < 0.01).
3. qPCR for apoptosis-related gene expression in HIEC-6 cells after 10 Gy irradiation with or without TT-2 treatment for 48 h (**p < 0.01).


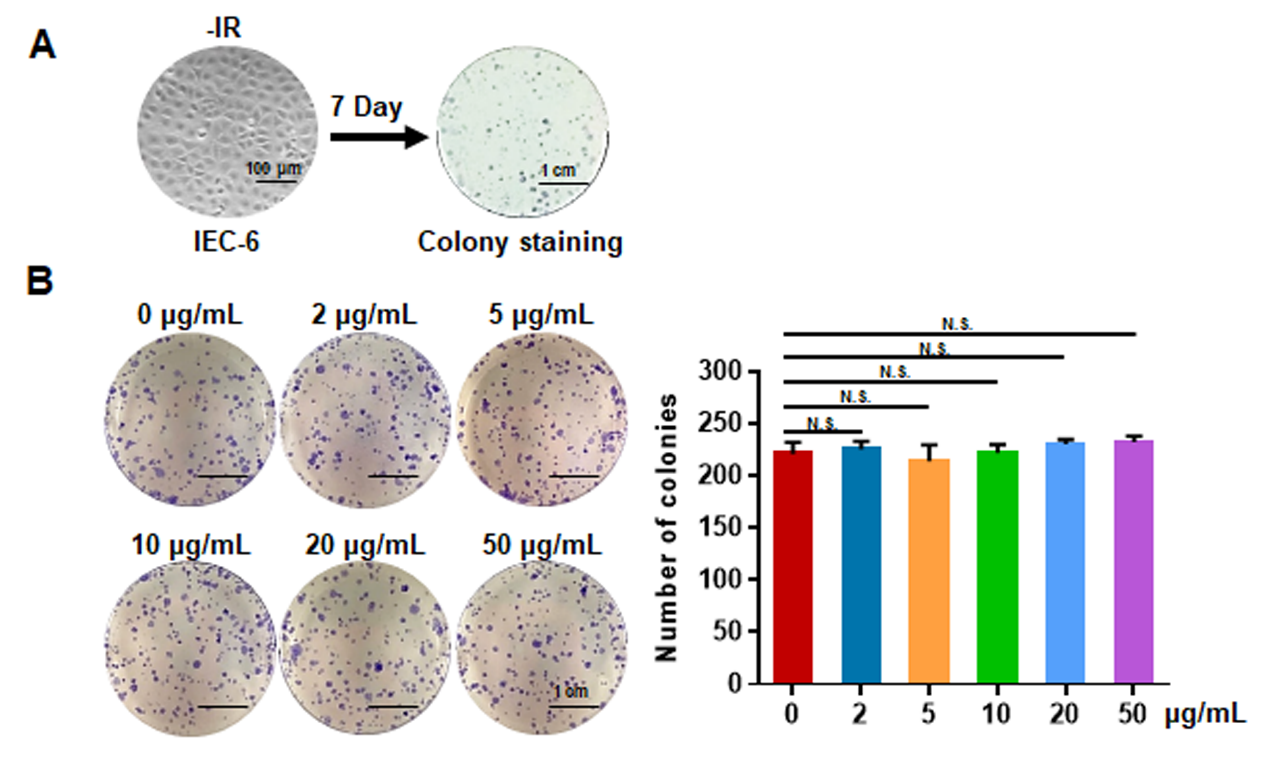
Supplementary Figure 2. Administration of TT-2 showed no effect on the unirradiated IEC-6 cells.

1. Schematic diagram of colony formation by unirradiated IEC-6 cells.
2. Representative colony image and colony numbers formed by unirradiated IEC-6 cells cultured with different concentrations of TT-2 (scale bar = 1 cm).


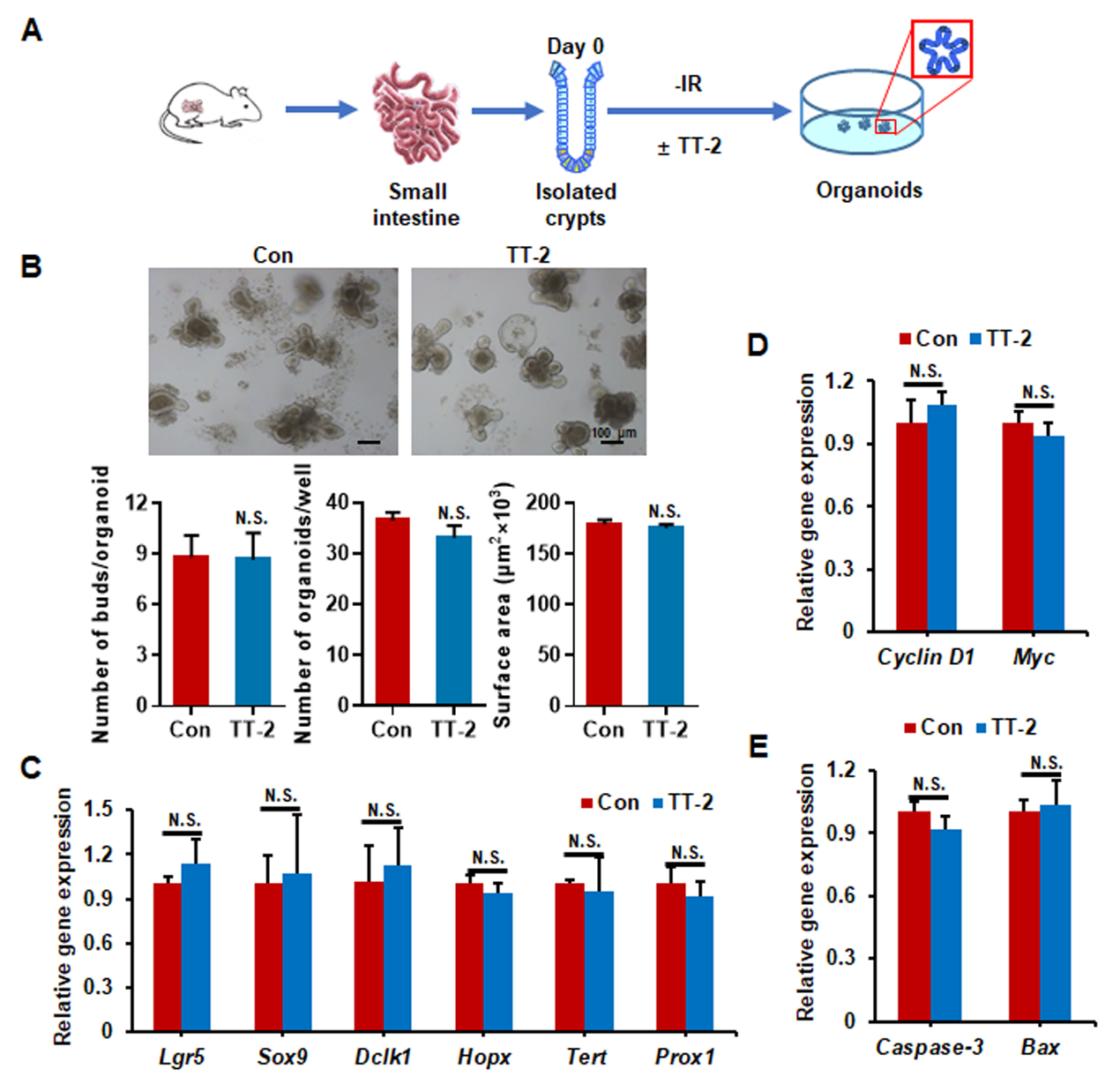
Supplementary Figure 3. TT-2 showed no significant effect on the unirradiated organoids

1. Schematic diagram of intestinal organoid culture and TT-2 treatment.
2. Representative phase contrast microscopic images and quantification analysis of intestinal organoid numbers per well, bud numbers, and surface area of each organoid under unirradiation condition with or without TT-2 addition (**p < 0.01, scale bar = 100 μm).
3. qPCR for ISC-related gene expression in unirradiated organoids with or without TT-2 treatment for 48 h.
4. qPCR for proliferation-related gene expression in unirradiated organoid with or without TT-2 treatment for 48 h.
5. qPCR for apoptosis-related gene expression in unirradiated organoids with or without TT-2 treatment for 48 h.
